# Pore formation in lipid membrane II: Energy landscape under external stress

**DOI:** 10.1038/s41598-017-12749-x

**Published:** 2017-10-02

**Authors:** Sergey A. Akimov, Pavel E. Volynsky, Timur R. Galimzyanov, Peter I. Kuzmin, Konstantin V. Pavlov, Oleg V. Batishchev

**Affiliations:** 1 A.N. Frumkin Institute of Physical Chemistry and Electrochemistry, Russian Academy of Sciences, 31/4 Leninskiy prospekt, Moscow, 119071 Russia; 2 National University of Science and Technology “MISiS”, 4 Leninskiy prospekt, Moscow, 119049 Russia; 3 Shemyakin-Ovchinnikov Institute of Bioorganic Chemistry, Russian Academy of Sciences, 16/10 Miklukho-Maklaya str., Moscow, 117997 Russia; 4 Federal Research and Clinical Center of Physical-Chemical Medicine, 1a Malaya Pirogovskaya, Moscow, 119435 Russia; 5 Moscow Institute of Physics and Technology, Institutsky lane 9, 141700 Dolgoprudniy, Russia

## Abstract

Lipid membranes are extremely stable envelopes allowing cells to survive in various environments and to maintain desired internal composition. Membrane permeation through formation of transversal pores requires substantial external stress. Practically, pores are usually formed by application of lateral tension or transmembrane voltage. Using the same approach as was used for obtaining continuous trajectory of pore formation in the stress-less membrane in the previous article, we now consider the process of pore formation under the external stress. The waiting time to pore formation proved a non-monotonous function of the lateral tension, dropping from infinity at zero tension to a minimum at the tension of several millinewtons per meter. Transmembrane voltage, on the contrary, caused the waiting time to decrease monotonously. Analysis of pore formation trajectories for several lipid species with different spontaneous curvatures and elastic moduli under various external conditions provided instrumental insights into the mechanisms underlying some experimentally observed phenomena.

## Introduction

Amphiphilic lipid matrix of biological membranes allows them to perform barrier function preserving integrity of cells and their compartments. Appearance of conductive transmembrane defects disrupts normal cell homeostasis leading to its death. On the other hand, controlled formation of membrane pores is necessary for wide range of biomedical and biotechnological applications such as cell electrotransfection^1^, electrofusion^2^, drug delivery^3^, etc. Controlling pore formation process requires detailed knowledge of all the possible mechanisms driving pore appearance, widening, and resealing, as well as the role of mechanochemical parameters of lipids in such processes.

Lipid membranes could be subjected to external stress, such as applied lateral tension or influence of electric field. Under certain conditions, these stimuli can lead to formation of transversal pores in the membrane. If we treat the lipid bilayer as an infinitely thin film without internal structure, according to the classical pore formation theory^4^, the energy of a cylindrically symmetric pore with the radius of *r* will consist of two contributing terms. The first term is proportional to the pore perimeter 2*πr* and characterized by the so-called line tension of pore edge, *γ*, which is an energetic parameter equal to the work performed to create a unit length of pore boundary. This term depends on internal membrane properties, such as its elastic parameters, so it is predominantly determined by the chemical structure of membrane-forming lipids^5–7^. The second contribution is proportional to the pore lumen area *πr* ^2^. It corresponds to the work of external forces, such as lateral tension, *σ*_0_. Thus, pore energy can be expressed as:

1 $$E(r)=2\pi r\gamma -\pi {r}^{2}{\sigma }_{0}.$$

It has a maximum at the critical radius of ${r}^{\ast }=\frac{\gamma }{{\sigma }_{0}}$, defining the energy barrier for the pore to become supercritical, whereafter irreversible rupture of the membrane occurs.

There are many experimental approaches designed for controllable formation of pores in lipid membranes. Most of them requires application of external stress to the membrane, such as lateral tension^8,9^, transmembrane electric potential^10,11^, or intense laser radiation^12^. Pore formation most probably starts from occurrence of a membrane hydrophobic defect in the form of a water-filled hydrophobic cylinder^13,14^ followed by bending and displacements of lipid molecules to avoid direct contact between lipid tails and polar environment along large area. Both the unfavorable contact of hydrophobic part of the membrane with water and the elastic deformations of the membrane at the pore edge allowing to avoid such contact impose energy penalties. In the classical theory of pore formation^4^, both contributions are attributed to one parameter, the line tension. The line tension is usually assumed to be independent on the pore radius, on the particular protocol of pore formation, and on the external stress applied to the membrane, e.g., on lateral membrane tension. However, these parameters appear to have a large impact on the line tension, wherefore the values measured in different experimental systems can vary by as much as an order of magnitude for the same lipid^11^.

In the previous article^15^ we have analyzed a complete trajectory of pore formation, starting from an intact bilayer through a hydrophobic defect to a hydrophilic pore in the stress-less membrane. The obtained trajectory is reversible and continuous in the sense that every state of the system is characterized by two continuous parameters — pore luminal radius and height of the hydrophobic belt at the pore equatorial plane. However, the system cannot spontaneously move along the trajectory from intact bilayer to large pores consuming energy of thermal fluctuations only, since the characteristic energies are on the order of tens of *k*_*B*_ *T*s (*k*_*B*_ *T*∼4·10^−21^ J)^15^. In this sense, the previous article reveals the energy landscape of a membrane with a transversal hydrophobic defect or a hydrophilic pore of various radii. Herein we are suggesting a mechanism of formation of a transverse pore in a lipid bilayer under external stress. Dependence of the trajectory on the applied lateral tension, transmembrane electric potential, and lipid composition of the membrane is analyzed.

## Continuum Theory of Elasticity

As in the previous article^15^, in the present work we treat membrane as a continuous liquid crystal medium, characterized by three moduli of elasticity: (1) splay, *B*; (2) tilt, *K*_*t*_; (3) lateral stretch/compression, *K*_*A*_. The state of the monolayer is described by a field of unit vectors **n**, known as directors, corresponding to time-averaged orientation of lipid molecules. The vector field is defined on the neutral surface, determined by unit normals **N** to it. At the neutral surface, the energy contributions from splay and lateral stretch/compression deformations are independent of each other. Splay is quantitatively characterized by divergence of the director along the neutral surface div(**n**). Tilt is characterized by deviation of the director from the normal at the given point of the neutral surface **t** = **n** − **N**. Lateral stretch/compression is characterized by change of the neutral surface area *a* relative to its area in the initial, non-deformed state *a*_0_, *α* = (*a* − *a*_0_)/*a*_0_. The elastic energy of a monolayer can thus be written as^16^:

2 $$W=\int \{\frac{B}{2}{(\text{div}({\bf{n}})+{J}_{0})}^{2}-\frac{B}{2}{J}_{0}^{2}+\frac{{K}_{t}}{2}{{\bf{t}}}^{2}+\frac{{K}_{A}}{2}{\alpha }^{2}+{\sigma }_{0}\}dS-{\sigma }_{0}{A}_{0},$$

where the integration is performed over the neutral surface of monolayer. Here *J*_0_ is the monolayer spontaneous curvature characterizing the preferred shape (curvature) of the monolayer in the absence of external forces and torques, *A*_0_ is the surface area of the monolayer in initial non-deformed equilibrium state; *σ*_0_ is the lateral tension of the monolayer. We assume the hydrophobic part of monolayers locally volumetrically incompressible, which is justified by large values of the volumetric compression moduli of membranes^17^. Within the adopted accuracy of approximation, local incompressibility condition reads^16^:

3 $${h}_{{\rm{c}}}=h-\frac{{h}^{2}}{2}\text{div}({\bf{n}})-h\alpha ,$$

where *h*_*c*_ and *h* are thicknesses of monolayer hydrophobic parts in the current and initial, non-deformed state, respectively.

**Figure 1:**
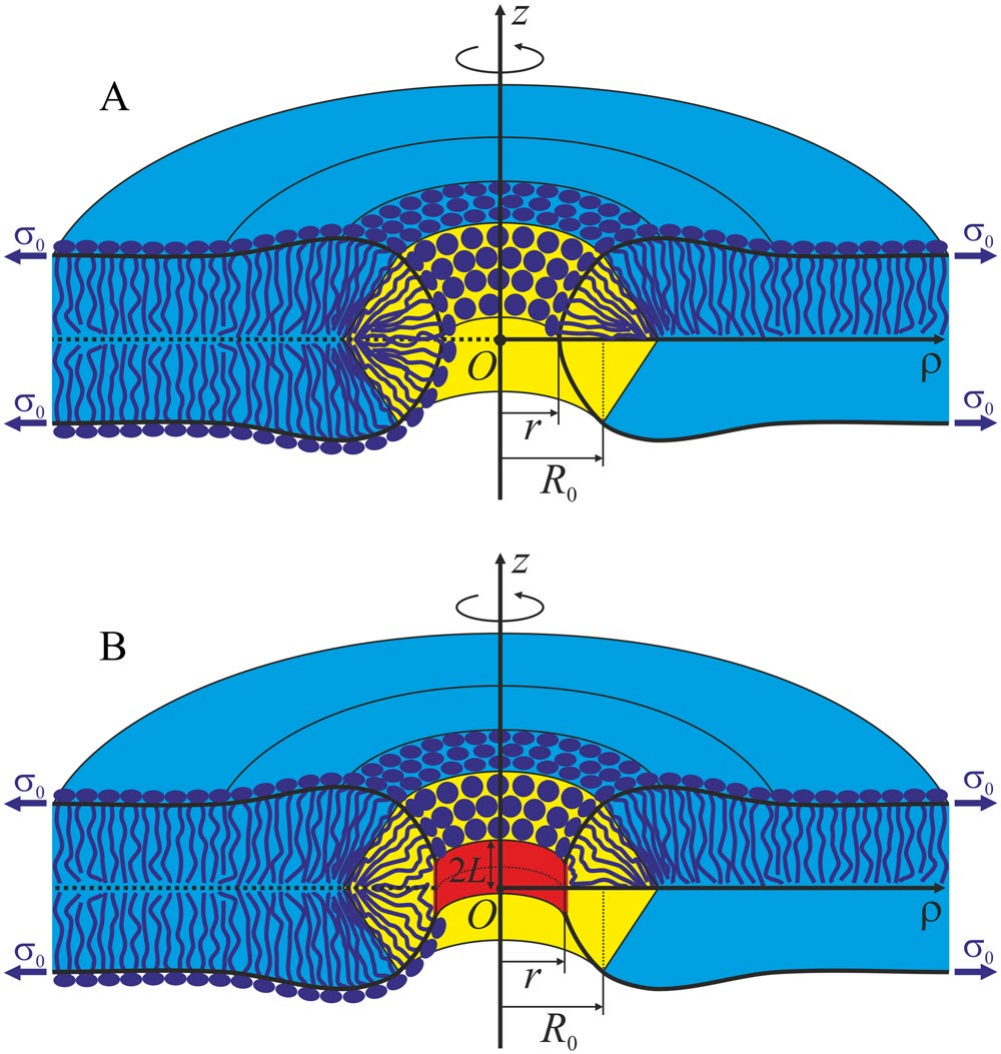
Schematic representation of membrane cross-section by a plane containing the rotational symmetry axis. The horizontal (bilayer) part of the membrane, in which the directors and normals are oriented approximately along the *Oz* axis is shown in blue; the vertical part where the directors and normals weakly deflect from the direction of the *Oρ* axis is highlighted in yellow. The parts are conjugated along two circles of equal radii *R*_0_. The pore radius is designated as *r*. (**A**) Hydrophilic pore; (**B**) hydrophobic defect. Cylindrical hydrophobic belt of the height 2 *L* and radius *r* is highlighted in red. Each monolayer is subjected to lateral tension *σ*_0_.

We use cylindrical coordinates {*O*, *z*, *ρ*} with the origin *O* in the point of intersection of the rotational symmetry axis with the mirror symmetry plane, *Oz* axis along the rotational symmetry axis and *Oρ* axis perpendicular to it. In order to reduce overestimation of the elastic energy resulting from application of linear theory of elasticity to highly deformed pore edge, we split the edge into two parts: almost horizontal bilayer, continuously conjugated with almost vertical monolayer along two circles, defined by coordinates {*R*_0_, ±*Z*_0_} (Fig. 1A).

It is assumed that hydrophilic pore is formed in the originally intact bilayer through an intermediate state referred to as a hydrophobic defect. We postulate it to consist of a horizontal bilayer region, vertical monolayer region, and cylindrical hydrophobic belt of the height of 2*L* and radius *r*, coaxial with *Oz* (Fig. 1B). The energy of water-filled hydrophobic cylinder is calculated in the refs^14,18^ based on Marcelja theory^19^ as:

4 $${W}_{h}=(4\pi rL){\sigma }_{h}\frac{{I}_{1}(\frac{r}{{\xi }_{h}})}{{I}_{0}(\frac{r}{{\xi }_{h}})},$$

where (4*πrL*) is the cylinder side surface area; *σ*_*h*_ is macroscopic lateral tension at the surface separating lipid tails and water; *ξ*_*h*_∼1 nm is characteristic length of hydrophobic interactions^20^; *I*_0_, *I*_1_ are Bessel functions of order zero and one, respectively.

Further, we have explicitly written Eqs (2), (3) for the horizontal bilayer and vertical monolayer parts. The resulting elastic energy functionals were minimized with respect to spatial distribution of deformations, resulting in corresponding Euler-Lagrange equations. The equations were solved analytically. The integration constants were obtained from the boundary conditions: 1) Directors and neutral surfaces were required to be continuous everywhere; 2) Deformations were required to decay at large distance from the pore edge. The remaining indefinite coefficients were found from the total energy minimum condition

5 $$W={W}_{b}+{W}_{m}+{W}_{h}{\textstyle \text{-}}\pi {r}^{2}(2{\sigma }_{0}),$$

where *W*_*b*_, *W*_*m*_, and *W*_*h*_ are elastic energies of horizontal bilayer, vertical monolayer, and energy of hydrophobic belt, respectively. Then the total free energy of the pore, Eq. (5), was minimized with respect to coordinates of conjugation of horizontal bilayer with vertical monolayer, *R*_0_ and *Z*_0_, by gradient descent method. It is worth pointing out that though application of Euler-Lagrange formalism to our model yields analytical expression for pore energy under given boundary conditions, the position of the boundary itself affects the resultant pore energy. Optimization of energy with respect to this parameter {*R*_0_, ±*Z*_0_} was performed numerically using gradient descent method, hence no analytical expression was obtained for the final optimized energy of the pore, and consequently for the line tension. The full details of calculations are presented in the previous article^15^.

Equation (5) defines free energy of a membrane with a pore of a radius *r*. The last term (−*πr* ^2^(2*σ*_0_)) corresponds to the work performed by the external forces (the lateral tension) and is hence negative. The first three terms (*W*_*b*_ + *W*_*m*_ + *W*_*h*_) correspond to the internal energy, i.e. the energy necessary to create the pore boundary. This energy related to the pore boundary perimeter is referred to as the line tension:

6 $$\gamma =\frac{{W}_{b}+{W}_{m}+{W}_{h}}{2\pi r}.$$

Note that the line tension implicitly depends on the lateral tension, since *γ* is mainly determined by the membrane elastic deformations at the pore edge, which depend on the applied lateral tension.

## Results and Discussion

### System parameters

The results obtained with the aid of continuum theory of elasticity will be illustrated for a generic model lipid, and three real lipids: 1,2-dioleoyl-sn-glycero-3-phosphocholine (DOPC), 1-palmitoyl-2-oleoyl-sn-glycero-3-phosphocholine (POPC), and 1,2-dimyristoyl-sn-glycero-3-phosphocholine (DMPC). The elastic parameters of model lipids are indicated by “*m*” index. The reference model lipid is assumed to have the following parameters: splay modulus (per monolayer) *B*_*m*_ = 8 *k*_*B*_ *T*; lateral stretch/compression modulus (per monolayer) *K*_*A*_^*m*^ = 100 mN/m; thickness of the hydrophobic part of the monolayer *h*_*m*_ = 2 nm, spontaneous curvature *J*_*m*_ = 0. Besides, the effects of spontaneous curvature are considered for the curvature values of *J*_*m*_ = −0.1 nm^−1^; +0.1 nm^−1^. Influence of other parameters on the energy landscape was analyzed in details in the previous article^15^.

For DOPC, the following values of elastic parameters are measured: splay modulus (per monolayer) *B* = 10.3 ± 1.2 *k*_*B*_ *T* ^21^; lateral stretch/compression modulus (per monolayer) *K*_*A*_ = 133 ± 9 mN/m^21^; thickness of the hydrophobic part of the monolayer *h* = 1.45 ± 0.02 nm^22^; spontaneous curvature *J*_*DOPC*_ = −0.091 ± 0.008 нм^−1^ (ref.^22^) or −0.11 nm^−1^ (ref.^23^); we use the value *J*_*DOPC*_ = −0.091 nm^−1^. The parameters measured for POPC are as follows: splay modulus (per monolayer) *B* = 11 *k*_*B*_ *T* ^21,24^; lateral stretch/compression modulus (per monolayer) *K*_*A*_ = 117 mN/m^21^; thickness of the hydrophobic part of the monolayer *h* = 1.46 nm^25^; spontaneous curvature *J*_*POPC*_ = −0.022 nm^−1^ (ref.^22^). The elastic parameters measured for DMPC are as follows: splay modulus (per monolayer) *B* = 6.8 *k*_*B*_ *T* ^21^; lateral stretch/compression modulus (per monolayer) *K*_*A*_ = 117 mN/m^21^; thickness of the hydrophobic part of the monolayer *h* = 1.37 nm^21^. There are no published results of measurement of DMPC spontaneous curvature. However, for the saturated lipid 1,2-dipalmitoyl-sn-glycero-3-phosphocholine (DPPC), the following value of spontaneous curvature is reported: *J*_*DPPC*_ = +0.068 nm^−1^ (ref.^22^). DMPC has smaller volume of the hydrophobic part of the molecules, so it should have a somewhat more positive spontaneous curvature. Therefore, we assumed that DMPC spontaneous curvature is *J*_*DMPC*_ = +0.075 nm^−1^. Equal tilt modulus of *K*_*t*_ = 40 mN/m (per monolayer) was assumed for all the lipids^26^. In ref.^20^, characteristic length of hydrophobic interactions *ξ*_*h*_ = 1 nm was experimentally determined. However, in these experiments the hydrophobic surface was rather rigidly bound to a solid support, restricting radial displacements possible for the hydrocarbon tails of the lipids forming the hydrophobic belt surface. Such displacements may cause *ξ*_*h*_ to increase. In the previous article^15^ we have demonstrated that the exact value of this parameter does not substantially influence the results of calculations. However, when considering the uncertainty of the numerical results obtained in the framework of our continuum model associated with the experimental errors of the measured elastic parameters, we carried out calculations for two values of the characteristic length of hydrophobic interactions: *ξ*_*h*_ = 1 nm and *ξ*_*h*_ = 1.5 nm. The surface tension at the interface between lipid tails and water was assumed at *σ*_*h*_ = 36 mN/m^27^.

We consider the range of membrane lateral tensions 0 < 2*σ*_0_ < 20 mN/m. Most durable lipid membranes can withstand for traceable time the lateral tension of about 10 mN/m^8,28^. However, if lateral tension linearly increasing in time is applied, membrane rupture can occur at higher tensions, up to 2*σ*_0_∼20 mN/m^8,28^.

### Trajectory of pore formation via hydrophobic defect

**Figure 2:**
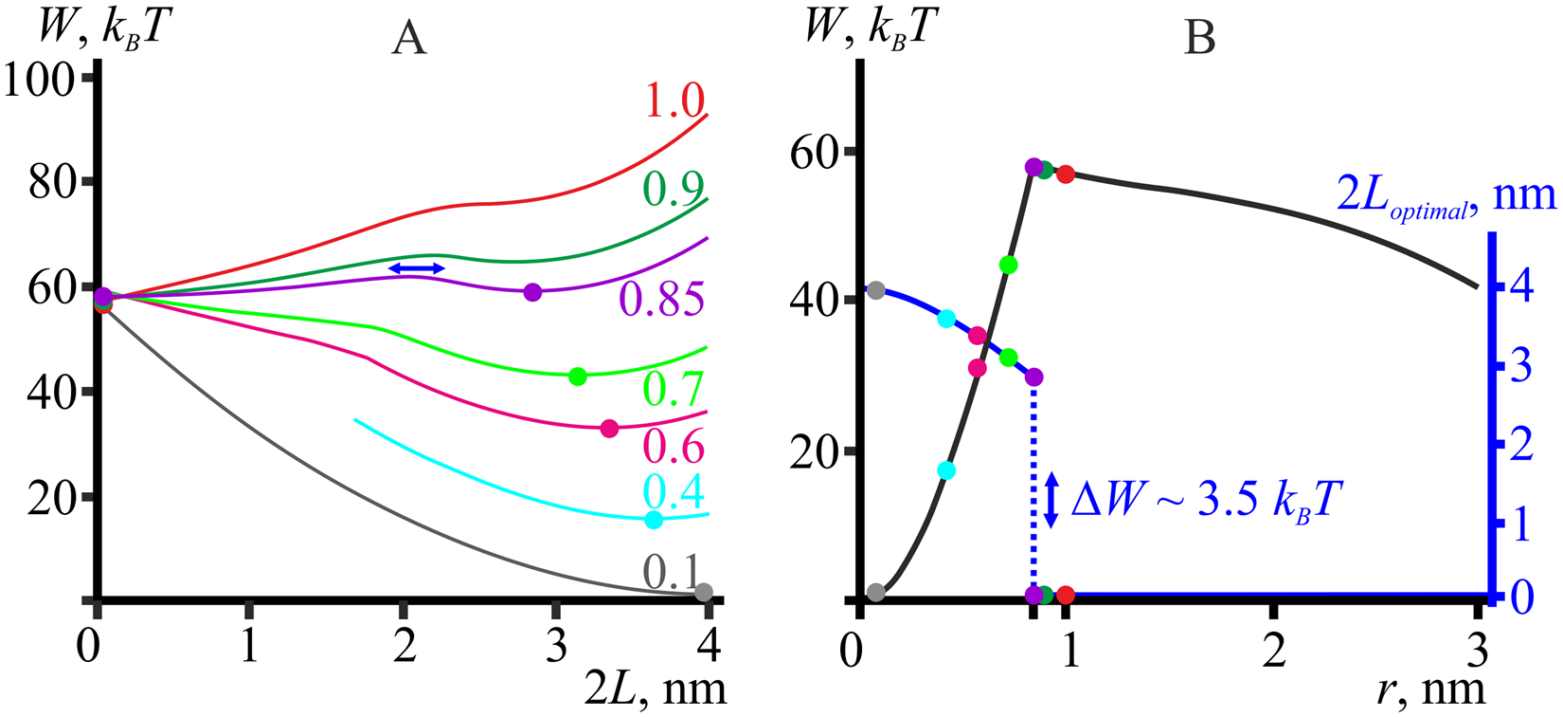
(**А**) Dependence of pore energy on the hydrophobic belt height, 2*L*, for the reference model lipid at different pore radii (specified near each curve in nanometers) for the membrane lateral tension 2*σ*_0_ = 10 mN/m. The elastic parameters of the reference model lipid are as follows: *B*_*m*_ = 8 *k*_*B*_ *T*, *h*_*m*_ = 2 nm, and *K*_*A*_^*m*^ = 100 mN/m. The dependencies *W*(*L*) have global minima marked by color circles. For the radius *r*∼0.85 nm (violet curve), pore energy *W*(*L*) has two minima with identical energies — at 2 *L* = 0 and at 2 *L* = 2.8 nm, separated by an energy barrier of about 3.5 *k*_*B*_ *T*. For *r* > 0.9 nm, the global energy minimum corresponds to 2 *L* = 0, i.e. to the hydrophilic pore. (**B**) Blue curve corresponding to the blue axis on the right: the dependence of optimal height of the hydrophobic belt, 2*L*_*optimal*_, on the pore radius. *L*_*optimal*_ is obtained from the positions of minima of the dependencies *W*(*L*) presented on the panel A and marked by color circles. Black curve corresponding to the black axis on the left: dependence of optimal pore energy on the radius *r* for the reference model lipid. The optimal pore energy is the energy at the minima of the dependencies *W*(*L*), presented on the panel A and marked by color circles.

The trajectory of pore formation in the membrane exposed to no external stress was analyzed in the previous article^15^. The algorithm of obtaining of the optimal trajectory of pore formation, *W*(*r*), at non-zero lateral tension is illustrated in Fig. 2 for the membrane lateral tension of 2*σ*_0_ = 10 mN/m. Briefly, we varied the half-height of the hydrophobic belt, *L*, for a given pore radius and plotted the dependencies of the pore energy *W*(*r*, *L*) on *L* (Fig. 2A). At each fixed radius, the energy *W*(*r*, *L*) had a global minimum with respect to *L*, with only one exception: at *r*∼0.85 nm the dependence *W*(*L*) has two minima of equal energy (∼58 *k*_*B*_ *T*) at 2 *L* = 0 and 2 *L* = 2.8 nm, separated by low energy barrier of about 3.5 *k*_*B*_ *T*, which implies a high frequency of transitions between the minima. Around this radius, transitions between a hydrophobic defect (2 *L* > 0) and a hydrophilic pore (2 *L* = 0) takes place. For the radii *r* ≥ 0.9 nm, the energy minima correspond to 2 *L* = 0, i.e. to the hydrophilic pore. Location of each minimum determined the optimal height of the hydrophobic belt, 2 *L*_*optimal*_, and the minimal energy value at the given pore radius. The optimal height of the hydrophobic belt thus depended on the pore radius (Fig. 2B, blue curve, blue axis on the right). This dependence, 2*L*_*optimal*_(*r*), determined the optimal trajectory of the pore formation: *W*(*r*) = *W*(*r*, *L*_*optimal*_(*r*)) (Fig. 2B, black curve, black axis on the left).

**Figure 3:**
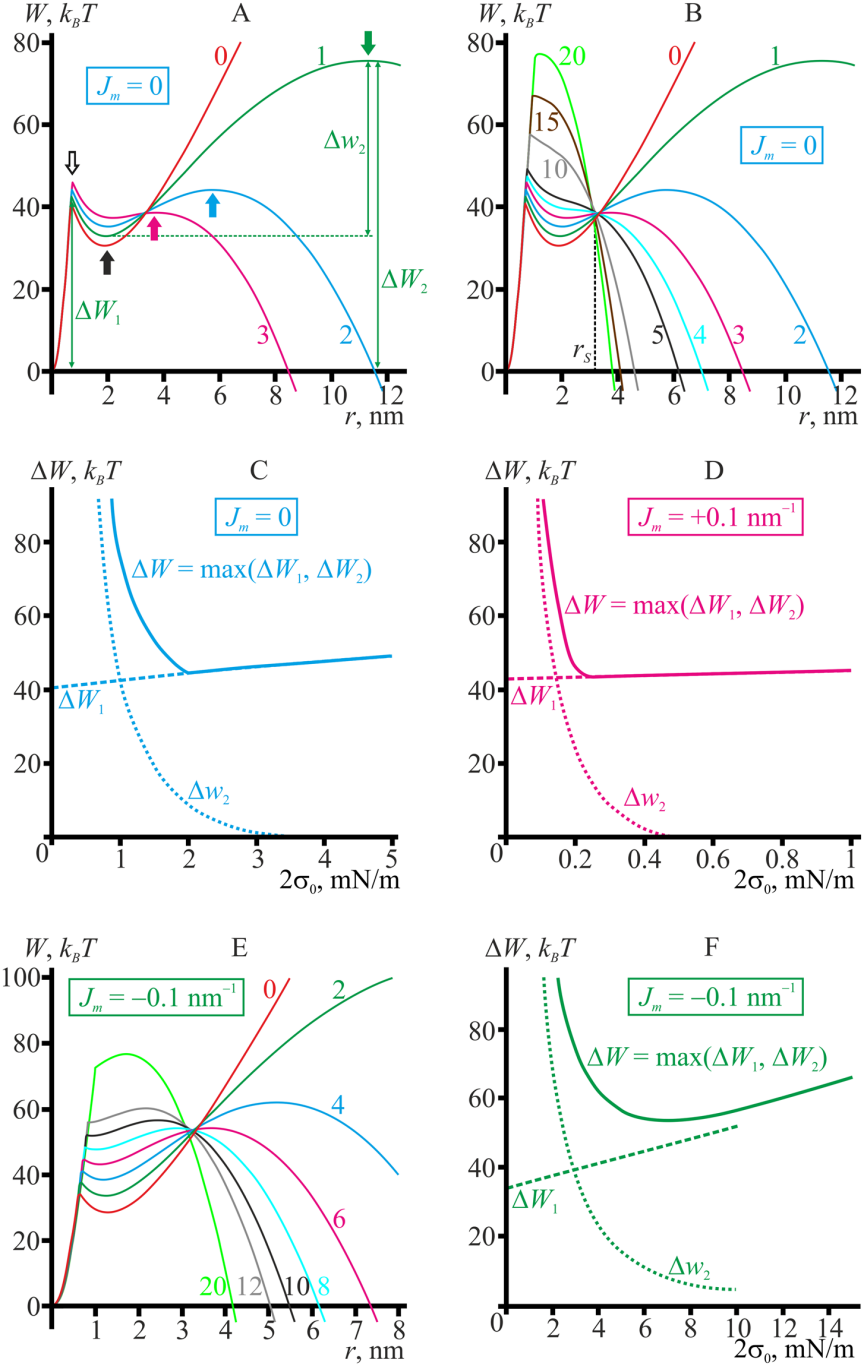
(**A**) Pore energy, *W*(*r*), optimized with respect to the height of the hydrophobic belt as a function of the pore radius calculated for the reference model lipid (*B*_*m*_ = 8 *k*_*B*_ *T*, *K*_*A*_^*m*^ = 100 mN/m, *h*_*m*_ = 2 nm, *J*_*m*_ = 0) under different values of lateral tension applied to the bilayer membrane, 2*σ*_0_ = 0–3 mN/m. The energy barrier Δ*W*_1_ at *r*∼1 nm, corresponding to transition from hydrophobic defect to hydrophilic pore, is indicated by a white arrow; the local minimum at *r*∼2 nm defining the metastable state is indicated by a black arrow; the energy barriers Δ*W*_2_ corresponding to transition to supercritical (infinitely growing) pore are indicated for each curve by an arrow of matching color. (**B**) Pore energy, *W*(*r*), for the membrane made of the reference model lipid under the lateral tension of 2*σ*_0_ = 0–20 mN/m. (**C**) Dependence of the pore formation energy barriers for the reference model lipid upon the membrane lateral tension, 2*σ*_0_. Δ*W*_1_ — the barrier for transition from hydrophobic defect to hydrophilic pore (dashed curve); Δ*w*_2_ — the barrier for transition from metastable state to supercritical pore (dotted curve); Δ*W* — the maximal (rate-limiting) energy barrier, Δ*W* = max(Δ*W*_1_, Δ*W*_2_). The barriers are schematically shown in the panel A for 2*σ*_0_ = 1 mN/m (green curve). For the lateral tensions 2*σ*_0_ > 2 mN/m, the metastable state at *r*∼2 nm as well as the second energy barrier vanish when the first energy barrier is higher than the second one (Δ*W*_1_ > Δ*W*_2_). Only the first energy barrier corresponding to transition of the hydrophobic defect to the hydrophilic pore remains in this range of lateral tensions; the maximum energy barrier thus coincides with the first energy barrier (dashed and solid curves coincide for 2*σ*_0_ > 2 mN/m). (**D**) Dependence of energy barriers of pore formation for the model lipid with positive spontaneous curvature, *J*_*m*_ = +0.1 nm^−1^, on the membrane lateral tension, 2*σ*_0_. (**E**) Pore energy, *W*(*r*), for the membrane made of the model lipid with negative spontaneous curvature, *J*_*m*_ = −0.1 nm^−1^, under the lateral tensions 2*σ*_0_ = 0–20 mN/m. The metastable state characterized by the local minimum of the pore energy at *r*∼1.5 nm vanishes for the lateral tension 2*σ*_0_ > 10 mN/m (compare black and grey curves), when the first energy barrier is lower than the second one (Δ*W*_1_ < Δ*W*_2_). The first barrier becomes just a “cusp” on the curve *W*(*r*). (**F**) Dependence of energy barriers of pore formation for the model lipid with negative spontaneous curvature, *J*_*m*_ = −0.1 nm^−1^, on the membrane lateral tension, 2*σ*_0_. In the entire range of lateral tensions, the first energy barrier (transition of the hydrophobic defect to the hydrophilic pore) is lower than the second one (transition to supercritical pore), Δ*W*_1_ < Δ*W*_2_, and thus the maximum energy barrier coincides with the second energy barrier, Δ*W* = Δ*W*_2_. For the lateral tensions 2*σ*_0_ > 10 mN/m the metastable state at *r*∼1.5 nm as well as the first energy barrier vanish. Only the second energy barrier remains in this range of the lateral tensions. The lateral tension values (per bilayer) in mN/m for each curve are specified on the plots in the same color as the corresponding curve in panels A, B, E.

Figure 3B presents optimal trajectories, *W*(*r*), of pore formation through hydrophobic defect for the reference model lipid at different values of lateral tension, 2*σ*_0_. At low but non-zero lateral tension values, 0 < 2*σ*_0_ < 3 mN/m, two energy barriers have to be crossed to form a supercritical pore of a large radius (Fig. 3A). The first energy barrier located at the pore radius of ∼1 nm, corresponds to transition from hydrophobic defect to hydrophilic pore (white arrow). After surmounting the barrier, the system enters a metastable state corresponding to the local energy minimum at *r*∼2 nm (black arrow). In the previous article^15^ the appearance of the minimum was attributed to the optimal mutual compensation of meridional (positive) and equatorial (negative) curvature of the pore boundary surface at this radius. Formation of a supercritical (large) pore, the energy of which monotonously decreases with increasing radius, requires surmounting the second energy barrier (Fig. 3A, green, blue, and magenta arrows). The barrier height referred to the ground state is designated as Δ*W*_2_; the height referred to the metastable state is designated as Δ*w*_2_ (Fig. 3A). At increasing lateral tension, both the critical radius (location of the top of the barrier) and the height of this second barrier decrease abruptly.

At the lateral tension 2*σ*_0_ ≈ 2 mN/m, the heights of barriers become equal, Δ*W*_1_ = Δ*W*_2_ (Fig. 3A, blue curve). For the reference model lipid, at the lateral tension of 2*σ*_0_ > 3.5 mN/m the local energy minimum at *r*∼2 nm and the second energy barrier vanish and only one energy barrier at *r*∼1 nm remains on the trajectory of formation of supercritical pore (Fig. 3B). The height of the remaining barrier, Δ*W*_1_, slowly monotonously increases with increasing lateral tension (Fig. 3C, dashed curve). The maximal, and thus the rate-limiting, energy barrier Δ*W* = max(Δ*W*_1_, Δ*W*_2_) on the trajectory of formation of the supercritical pore (*r* → ∞) from the intact bilayer (*r* = 0) coincides with Δ*W*_2_ for 2*σ*_0_ < 2 mN/m, and with Δ*W*_1_ for 2*σ*_0_ > 2 mN/m (Fig. 3C, solid line). Since Δ*W*_2_ decreases with growing lateral tension, while Δ*W*_1_ increases, the maximal energy barrier depends non-monotonously on the lateral tension, having the minimum at 2*σ*_0_∼2 mN/m for the reference model lipid (Fig. 3C). Thus, according to our analysis the probability of formation of a supercritical pore should also non-monotonously depend on the lateral tension applied to the membrane. At zero lateral tension, pore energy is monotonously growing at large radii in an approximately linear manner as 2*πrγ*_0_, so that no supercritical pores can be formed (Fig. 3A, red curve). At low lateral tension, the height of the first energy barrier is ∼40 *k*_*B*_ *T* for the reference model lipid. According to the estimates made in ref.^27^, such a barrier can be crossed at the expense of thermal fluctuations within ∼1 minute, and the system can get into an energy minimum at *r*∼2 nm (Fig. 3A). However, at low lateral tension the second energy barrier is so high (120 *k*_*B*_ *T* for 2*σ*_0_ = 0.5 mN/m) that it cannot be realistically crossed through thermal fluctuations. In this case, even if the system gets into the energy minimum at *r*∼2 nm, the hydrophilic pore is most likely to close (*r* → 0) since the energy barrier for this process is much lower (10 *k*_*B*_ *T* versus 120 *k*_*B*_ *T* for 2*σ*_0_ = 0.5 mN/m). At higher lateral tensions, the height of the first energy barrier (*r*∼1 nm) corresponding to transition of hydrophobic defect to hydrophilic pore grows slightly, but the height of the second barrier decreases rapidly, completely vanishing at 2*σ*_0_∼3.5 mN/m (Fig. 3C, dotted curve). Decrease of the height of the second barrier is accompanied by growth of the first, initially considerably smaller barrier. Thus, it takes the system progressively longer to get into the energy minimum at *r*∼2 nm, but the chance of crossing the second barrier within the experimentally observable timescale increases. Overall, the increase of the first barrier in the lateral tension range of 2*σ*_0_∼0–2 mN/m is relatively small, and the total probability of supercritical pore formation gets higher. With further growth of lateral tension, 2*σ*_0_ > 3.5 mN/m, the second energy barrier vanishes and the height of the first barrier keeps monotonously increasing, resulting in smaller probability of supercritical pore formation (higher average waiting time). Thus, the average time of formation of a supercritical pore has a local minimum at certain lateral tension. For the reference model lipid, the minimum corresponds to the lateral tension of about 2*σ*_0_∼2 mN/m, at which the first energy barrier (Δ*W*_1_∼45 *k*_*B*_ *T*) can still be crossed via thermal fluctuations, while the height of the second barrier is already small enough, Δ*w*_2_∼10 *k*_*B*_ *T* (Fig. 3C).

The dependencies of the pore energy on the pore radius, *W*(*r*), built for different values of the membrane lateral tension, intersect in the vicinity of some radius *r*_*S*_∼3.2 nm (Fig. 3B). For smaller radii, *r* < *r*_*S*_, the curves *W*(*r*) grow up as the lateral tension increases, while for *r* > *r*_*S*_ the curves go down. The energy *W*(*r*_*S*_) appears to be independent on the lateral tension, since this point does not shift upon variation of the lateral tension. As the first energy barrier is located at *r*∼1 nm < *r*_*S*_, the barrier height increases with growing 2*σ*_0_ (Fig. 3C). The second energy barrier vanishes before the critical radius (location of the top of the barrier) becomes smaller than *r*_*S*_, i.e. the critical radius always exceeds *r*_*S*_ for the reference model lipid. Thus, the height of the second energy barrier monotonously decreases for growing lateral tension (Fig. 3C). The physical reason for existence of the radius *r*_*S*_ of intersection of the dependencies *W*(*r*) calculated for different lateral tensions is discussed below, in the section “Pore line tension depends on the lateral tension”.

Non-zero spontaneous curvature does not qualitatively change the energy landscape of supercritical pore formation for the model lipid (Fig. 3C,D,F). In case of positive spontaneous curvature, (Fig. 3D) the height of the second barrier decreases very rapidly with increasing lateral tension, and vanishes entirely already at 2*σ*_0_∼0.5 mN/m (Fig. 3D, dotted curve). For 2*σ*_0_ > 0.25 mN/m the first energy barrier is higher than the second barrier, thus the maximum barrier coincides with the first one, Δ*W* = Δ*W*_1_. (Fig. 3D, dashed line coincides with the solid line for 2*σ*_0_ > 0.25 mN/m). The optimal lateral tension for pore formation in the membrane made of the model lipid with *J*_*m*_ = +0.1 nm^−1^ is about 2*σ*_0_ = 0.25 mN/m, as at this lateral tension the maximal energy barrier has a local minimum.

For negative spontaneous curvatures, the second barrier decreases much slower with the applied lateral tension (Fig. 3F), whereas the pore energy at the value of *r*∼1.5 nm corresponding to energy minimum grows rather rapidly. The first energy barrier (transition of the hydrophobic defect to the hydrophilic pore) is lower than the second one (transition to supercritical pore), Δ*W*_1_ < Δ*W*_2_ in the entire range of lateral tensions, and thus the maximum energy barrier coincides with the second energy barrier, Δ*W* = Δ*W*_2_. At 2*σ*_0_ > 10 mN/m, the local energy minimum corresponding to *r*∼1.5 nm vanishes, and the first barrier becomes just a “cusp” on the curve *W*(*r*) (compare grey and black curves in the Fig. 3E). Similar “cusp” on the *W*(*r*) energy dependence is observed in MD studies of the pore formation^29^, and is considered in the recent experimental studies of the pore formation kinetics^28^. For 2*σ*_0_ > 7 mN/m the critical radius (location of the second energy barrier) becomes smaller than the radius of intersection of *W*(*r*) curves (about 3.3 nm for the model lipid with *J*_*m*_ = −0.1 mN/m), and thus the barrier starts to grow with increasing lateral tension. The optimal lateral tension for pore formation in the membrane made of the model lipid with *J*_*m*_ = −0.1 nm^−1^ is about 2*σ*_0_ = 7 mN/m as at this lateral tension the maximal energy barrier has a local minimum (Fig. 3F).

The absolute and relative contributions to the pore edge energy are summarized in Table 1 for the model lipid with *J*_*m*_ = 0 and for the membrane lateral tensions of 2*σ*_0_ = 2 mN/m and 2*σ*_0_ = 4 mN/m. In Table 1, we only take into account the energy of the pore boundary; the term −*πr* ^2^ *σ*_0_ (Eq. (5)) proportional to the area of the pore lumen is neglected. At small radii (0.2 and 0.5 nm in Table 1), at the stage of hydrophobic defect, the surface tension at the interface between water and lipid tails, *σ*_*h*_, is small in accordance with Marcelja theory^19^. As a result, hydrophobic energy is a predominant contributor (>90%) to the total energy, as the deformation leading to its increase is “the softest mode”. However, *σ*_*h*_ rapidly increases with growing radius, leading to vanishing of the hydrophobic belt for *r* > 0.7 nm. At the radii *r* > 1 nm the softest deformational modes are splay and lateral tension resulting in comparable relative contributions to the pore edge energy, which are substantially larger than contributions of deformations of tilt and lateral stretch/compression (Table 1).

**Table 1:** Contributions of different deformational modes to the total energy of the pore boundary in the membrane made of the reference model lipid (*B*_*m*_ = 8 *k*_*B*_ *T*, *K*_*A*_^*m*^ = 100 mN/m, *h*_*m*_ = 2 nm, *J*_*m*_ = 0).

Pore radius, *r*, nm	0.2	0.5	1	3.5	10	200
Splay energy, *k*_*B*_ *T*/%	0.02/0.54%	0.85/3.38%	32.1/76.7%	32.6/55.8%	105/58.9%	2668/64.4%
	0.04/0.45%	0.68/2.67%	31.2/66.7%	33.0/43.1%	107/47.2%	2730/53.6%
Tilt energy, *k*_*B*_ *T*/%	0.05/1.11%	1.05/4.16%	1.85/4.44%	5.14/8.79%	16.0/9.0%	336/8.11%
	0.04/0.99	0.90/3.53%	1.97/4.14%	5.07/6.63%	15.6/6.88%	331/6.51%
Stretching energy, *k*_*B*_ *T*/%	0.01/0.22%	0.39/1.55%	1.98/4.73%	2.55/4.36%	7.50/4.21%	166/4.00%
	0.01/0.20%	0.34/1.35%	2.24/4.71%	2.80/3.66%	7.80/3.43%	168/3.30%
Hydrophobic energy, *k*_*B*_ *T*/%	4.34/97.97%	22.7/90.1%	0	0	0	0
	4.35/98.11%	23.1/91.1%				
Tension energy, *k*_*B*_ *T*/%	0.01/0.22	0.21/0.85%	5.90/14.1%	18.2/31.1%	49.8/27.9%	971/23.5%
	0.01/0.26	0.34/1.35%	11.7/24.5%	35.6/46.6%	96.5/42.4%	1861/36.6%
Total energy, *k*_*B*_ *T*	4.36	24.8	41.8	58.4	178.2	4140
	4.3	24.6	47.6	76.4	227.2	5091

The upper line in each cell corresponds to the membrane lateral tension 2*σ*_0_ = 2 mN/m; the lower line — to 2*σ*_0_ = 4 mN/m.

From Table 1 it is seen that at large radii higher values of the lateral tension disturb spatial distribution of deformations to greater extent, leading to somewhat increased absolute values of splay energy at decreasing relative contribution of this deformation (compare the upper and the lower lines in the cells corresponding to splay deformation). Increase of the lateral tension from 2*σ*_0_ = 2 mN/m to 2*σ*_0_ = 4 mN/m results in almost proportional growth of absolute value of the work needed to be performed against the lateral tension (compare the upper and the lower lines in the cells corresponding to tension energy).

### Tension-induced pore formation in DOPC, POPC, and DMPC membranes

**Figure 4:**
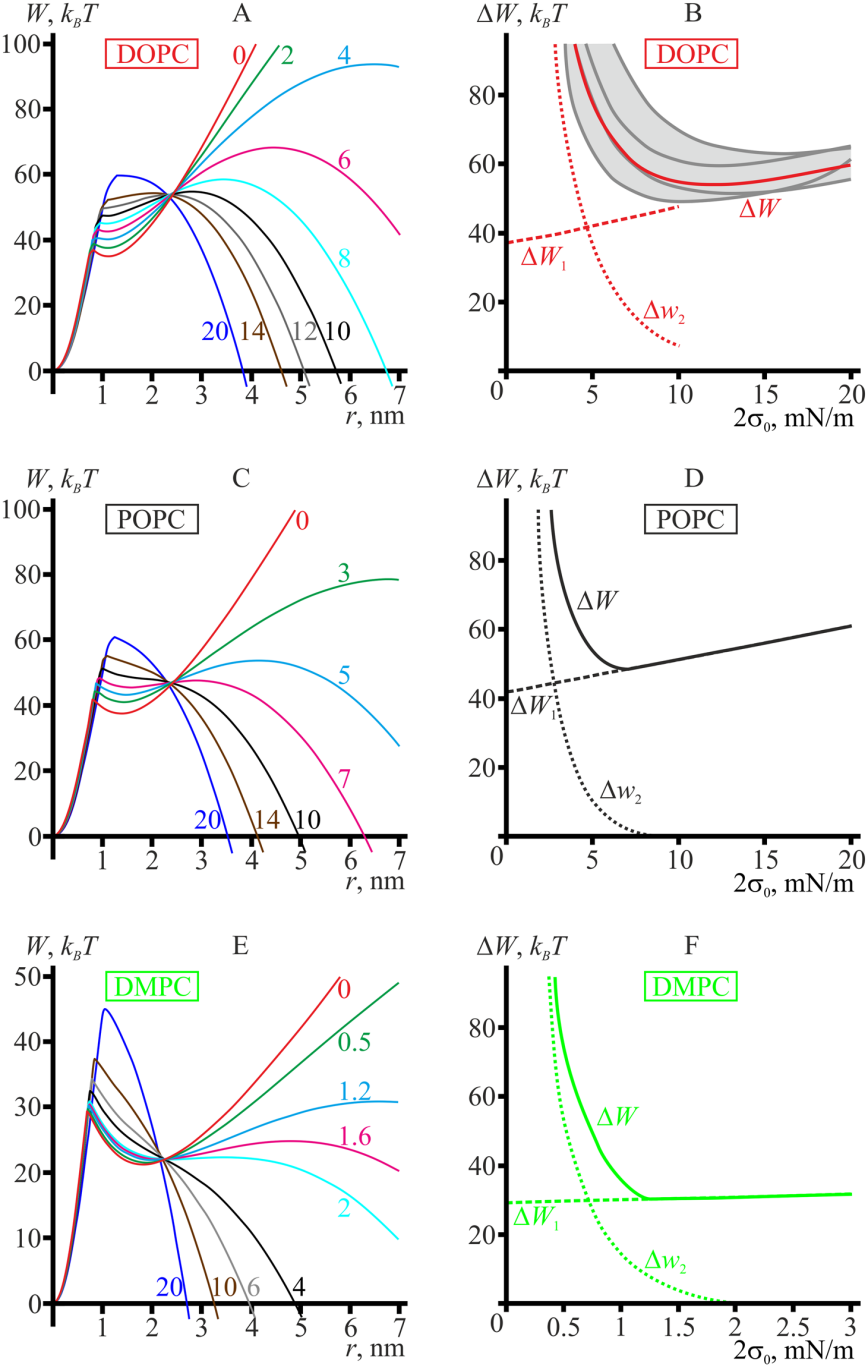
Pore energy, *W*(*r*), optimized with respect to the height of the hydrophobic belt as a function of the pore radius, and the dependence of energy barriers of pore formation on the membrane lateral tension, 2*σ*_0_, calculated for: DOPC (**A**,**B**); POPC (**C**,**D**); DMPC (**E**,**F**). The energy barriers Δ*W*_1_, Δ*w*_2_, Δ*W* are schematically illustrated in Fig. 3A. In panel B, the dark grey curves illustrate the dependencies of the rate-limiting energy barrier for pore formation, Δ*W*, on the applied lateral tension for various experimentally determined sets of elastic parameters. The dependencies were obtained taking into account the confidence interval of the elastic parameters as indicated in the Section “System parameters”. The curves provide bounding estimates for Δ*W*(2*σ*_0_) dependencies (light grey area). The lateral tension values (per bilayer) in mN/m for each curve are specified on the plots in the same color as the corresponding curve in panels A, С, E.

Optimal trajectories, *W*(*r*), of pore formation through hydrophobic defect, as well as energy barriers at different values of lateral tension, 2*σ*_0_, for DOPC, POPC, and DMPC membranes are presented in Fig. 4.

The dependencies of optimal trajectories on the applied lateral tension are qualitatively similar for the reference model lipid and POPC, for the model lipid with negative spontaneous curvature and DOPC, for the model lipid with positive spontaneous curvature and DMPC, respectively. At modest lateral tensions, there are two energy barriers on the pathway of formation of a large pore. The first barrier located at *r*∼1 nm corresponds to the transition from a hydrophobic defect to a hydrophilic pore. The second energy barrier corresponds to expansion of a hydrophilic pore to a supercritical one; its top position (the critical radius) strongly depends on the applied lateral tension. For each of the three lipids there is a certain radius *r*_*S*_, near which the trajectories *W*(*r*) for different lateral tensions intersect (compare Figs 3A,B,E and 4A,C,E). It means that the energy of the pore of the radius *r*_*S*_ is independent of lateral tension. For POPC and DMPC, as the lateral tension increases, the first energy barrier grows, while the second barrier gradually decreases and completely vanishes at 2*σ*_0_ = 8 mN/m for POPC and at 2*σ*_0_ = 2 mN/m for DMPC. The maximal rate-limiting energy barrier coincides with Δ*W*_2_ for small lateral tensions, and with Δ*W*_1_ for large lateral tensions (Fig. 4D,F). As Δ*W*_2_ decreases and Δ*W*_1_ increases with growing 2*σ*_0_, the maximal energy barrier proves to be a non-monotonous function of lateral tension, having a minimum at about 2*σ*_0_ = 7 mN/m for POPC and 2*σ*_0_ = 1.3 mN/m for DMPC (solid curves in Fig. 4D,F). These optimal values for pore formation for POPC and DMPC are quite close to the values of the quasi-statical (slowly growing) lateral tension, at which pores are most frequently formed in giant unilamellar vesicle (GUV) membranes made of 1-stearoyl-2-oleoyl-sn-glycero-3-phosphocholine and of diC13:0 lipid, as determined in the work ref.^8^. The minimal rate-limiting energy barriers of pore formation are 48 and 30 *k*_*B*_ *T* for POPC and DMPC, respectively (Fig. 4D,F).

For DOPC, the energy landscape of pore formation under applied lateral tension is qualitatively similar to that for the model lipid with negative spontaneous curvature (compare Figs 4A,B and 3E,F). Negative spontaneous curvature leads to relatively high line tension of the pore. In this case the local minimum of the pore energy at *r*∼1.5 nm is quite shallow even at zero lateral tension (Fig. 4A, red curve), and its depth progressively decreases at increasing lateral tension, as the whole curve *W*(*r*) grows for *r* < *r*_*S*_ (Fig. 4A). For DOPC, the local minimum and the first energy barrier completely vanish at 2*σ*_0_∼10 mN/m; the first barrier becomes just a “cusp” on the curve *W*(*r*) (Fig. 4A, grey, brown, and dark blue curves). Similar disappearance of the first energy barrier with formation of a “cusp” on the *W*(*r*) dependence is considered in the paper by E. Evans and B.A. Smith^28^. Here we attribute such behavior mostly to negative spontaneous curvature of the membrane-forming lipid. As the lateral tension increases, the second barrier as well as the critical radius decrease, and the critical radius becomes smaller than *r*_*S*_ for 2*σ*_0_ > 12 mN/m (Fig. 4A, grey, brown, and dark blue curves). Thereafter, the second barrier starts to grow with further increase of lateral tension (Fig. 4B). This leads to non-monotonous dependence of the maximal energy barrier of pore formation, Δ*W*, on the applied lateral tension; the barrier is minimal and equal to ∼54 *k*_*B*_ *T* at 2*σ*_0_∼12 mN/m (Fig. 4B, solid red curve).

The energy barriers for pore formation strongly depend on the elastic properties of the particular lipid (compare Fig. 4B,D,F). Evidently, experimentally determined elastic parameters have some uncertainties. In the Section “System parameters”, the parameters with corresponding uncertainties are presented for DOPC. We have analyzed the effect of the parameter uncertainties on the height of the rate-limiting energy barrier for DOPC by combining different limiting values of the elastic parameters (the upper and the lower limit of the experimental confidence interval) to distinct sets and calculating the dependence of the rate-limiting energy barrier for pore formation, Δ*W*, on the membrane lateral tension, 2*σ*_0_. The curves obtained for different sets of the elastic parameters envelope the area of possible Δ*W*(2*σ*_0_) dependencies (Fig. 4B, dark grey curves restricting the grey zone around the red curve). In the considered range of the elastic parameters, the optimal lateral tension for pore formation and the minimal height of the rate-limiting energy barrier, Δ*W*, vary in the range from 2*σ*_0_ = 10 mN/m, Δ*W* = 49 *k*_*B*_ *T* to 2*σ*_0_ = 16 mN/m, Δ*W* = 63 *k*_*B*_ *T*.

### Pore line tension depends on the lateral tension

**Figure 5:**
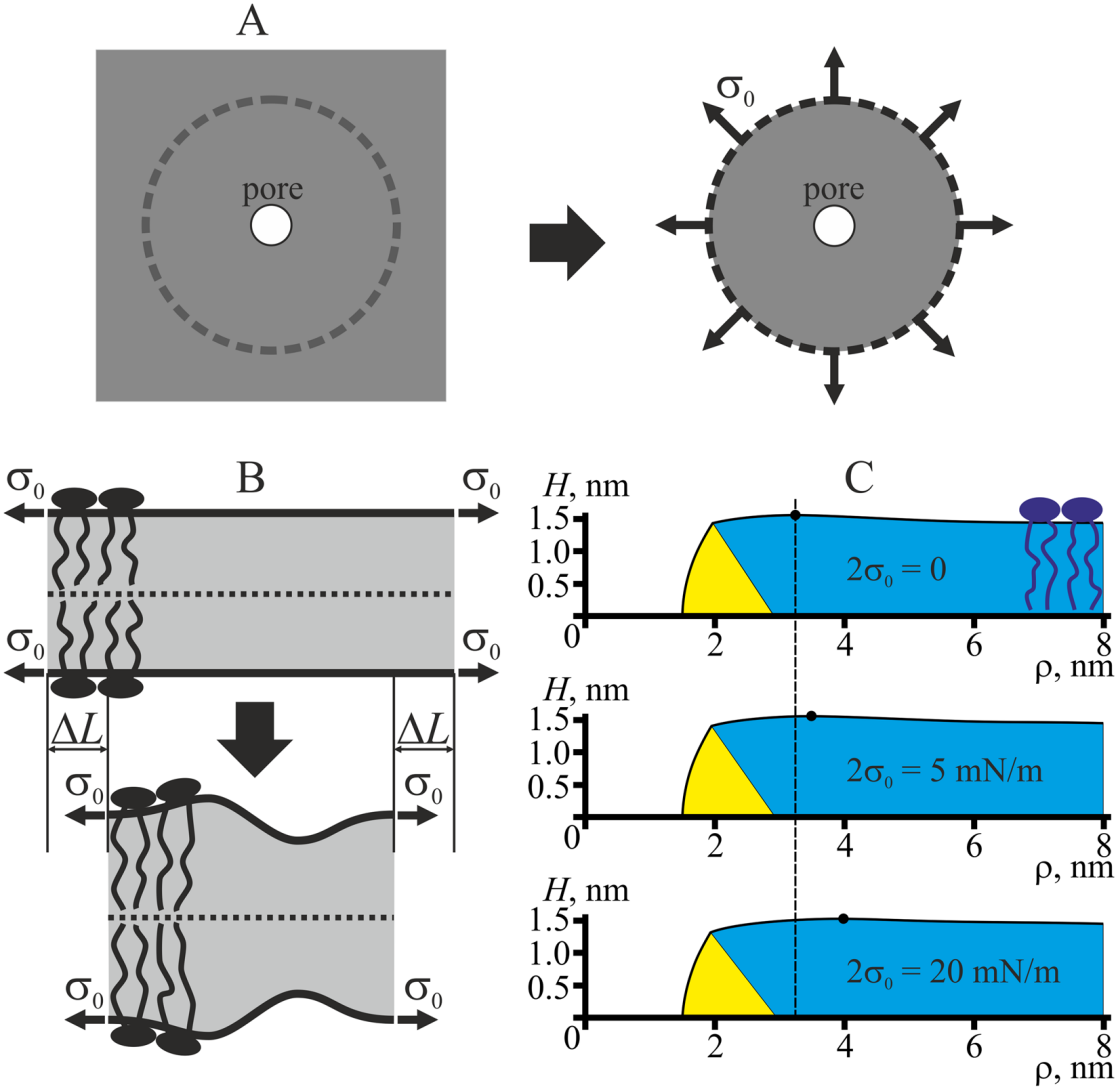
Membrane lateral tension contributes to elastic deformation energy. (**А**) For an isolated fragment of the membrane with the pore, lateral tension is viewed as a force distributed along the boundary of the fragment. (**B**) Membrane deformation requires additional work to be performed against the lateral tension, ∼*σ*_0_Δ*L*, even if the neutral surface area of the monolayers remains unchanged. (**C**) Membrane surface shape, *H*(*ρ*), in the vicinity of a pore with the radius of *r* = 1.5 nm for DOPC under different lateral tensions of the membrane, 2*σ*_0_. From top to bottom: *σ*_0_ = 0, 2*σ*_0_ = 5 mN/m, 2*σ*_0_ = 20 mN/m. Only the upper half of the membrane (above the equatorial plane) is shown due to mirror symmetry of the system. Solid black circles indicate the position of the maximum of *H*(*ρ*); the vertical dashed line passes through the maximum at zero lateral tension.

A somewhat counterintuitive effect of the lateral tension on the dependence of pore energy upon its radius, *W*(*r*), can be explained from consideration of the lateral tension as a force acting perpendicularly to a certain line on the neutral surface of a monolayer relative to the length of the line^30^. From the physical standpoint, membrane can be cut along a certain closed line around the pore at sufficiently large distance from its boundary and the part of the membrane outwards of the line can then be substituted with distributed force acting on this section line, i.e. with the lateral tension (Fig. 5А).

**Figure 6:**
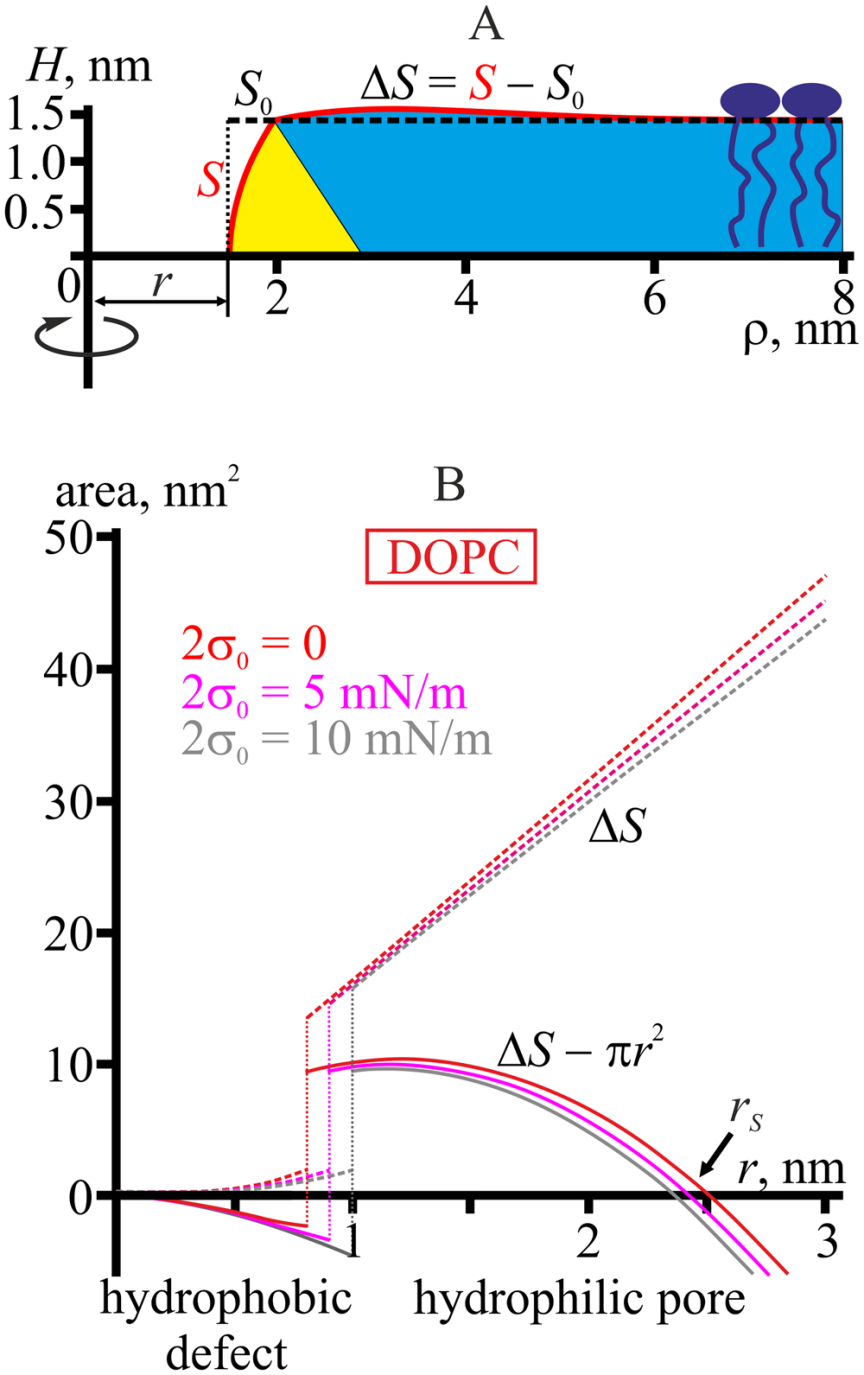
Balance of a monolayer neutral surface area at the pore boundary in DOPC membrane. (**A**) The actual neutral surface area, *S* (red curve), of the pore of the radius *r*. *S*_0_ denotes the initial (projected) area of the monolayer neutral surface in undeformed state (horizontal dashed line). (**B**) Dashed curves — dependence of the additional area pulled from lipid reservoir due to monolayer deformations at the pore edge, Δ*S*, on the pore radius, *r*. For the hydrophilic pore (*r* > 1 nm) the dependence is almost linear. Solid curves — dependence of the difference of the additional area pulled from lipid reservoir due to monolayer deformations at the pore boundary, Δ*S*, and the luminal area of the pore, *πr* ^2^, on the pore radius, *r*. For small hydrophilic pore (1 nm < *r* < 2.5 nm) the difference is positive, Δ*S* − *πr* ^2^ > 0, meaning that the additional work against the lateral tension should be performed in order to create the pore boundary. For larger pore radius, Δ*S* − *πr* ^2^ < 0, meaning that the lateral tension favors pore expansion. In the vicinity of the radius *r*_*S*_∼2.5 nm, Δ*S* − *πr* ^2^ = 0. The dependencies are built at different lateral tensions: 2*σ*_0_ = 0 (red curves), 5 mN/m (magenta curves), 10 mN/m (grey curves). Vertical dotted lines correspond to transition of the hydrophobic defect to the hydrophilic pore.

Lateral tension contributes to the elastic deformation energy due to local increase of monolayer surface area compared to its area in non-deformed state (projection area). If the extension of the membrane fragment around the pore can be neglected, deformation should cause a shift of the section line towards the pore boundary by a certain distance Δ*L*, which requires additional work of ∼*σ*_0_Δ*L* to be performed against lateral tension forces (Fig. 5B). Thus, after pore formation the energy of the system is reduced by the amount of work *σ*_0_ *πr* ^2^ performed by the external forces in the process and is increased by the amount of work performed by the system against the external forces in order to increase the neutral surface area of monolayers by Δ*S* = *S* − *S*_0_ from the initial area (projection area), *S*_0_, to the actual area, *S*, that is *σ*_0_Δ*S*. (Fig. 6A).

Overall contribution of lateral tension into the pore energy can be expressed as *σ*_0_Δ*S* − *σ*_0_ *πr* ^2^ = *σ*_0_(Δ*S* − *πr* ^2^). The increment Δ*S* is roughly proportional to the pore perimeter 2*πr*, i.e., linear with respect to the radius (Fig. 6B, dashed curves), whereas the luminal area depends quadratically on it. In a hydrophilic pore, at small radii, Δ*S* − *πr* ^2^ > 0 (Fig. 6B, solid curves), so that for the hydrophilic pore to be formed, additional monolayer surface area has to be created and hence certain work has to be performed against the lateral tension. This is the origin of the increase of system energy at small pore radius (*r*∼1.5 nm) under increasing lateral tension (Fig. 3), and of the dependence of the membrane surface shape on the lateral tension (Fig. 5C). For large pore radii Δ*S* − *πr* ^2^ < 0 (Fig. 6B, solid curves), hence lateral tension facilitates further growth of the pore. For the assumed model of the pore boundary structure, Δ*S* = *πr* ^2^ at *r*_*S*_∼2.5 nm for DOPC (Fig. 6B), where the curves *W*(*r*) of pore energy as a function of its radius intersect for different values of lateral tension (see Fig. 4A). At the stage of hydrophobic defect, the lumen radius is relatively small, and the term *πr* ^2^ is negligible. At the same time, membrane area, increased by deformations, can be adjusted by varying the height of the hydrophobic belt at the given radius. For this reason, the energy of the hydrophobic defect is almost independent on the lateral tension up to the tension values as high as 2*σ*_0_∼20 mN/m (Figs 3B,E and 4A,C,E).

The line tension is the energy (minimal work) necessary to create pore boundary of unit length. Under the applied lateral tension, this energy should increase by the additional work performed against the lateral tension in order to pull additional area, Δ*S*, of the neutral surface towards the boundary (Fig. 6B, dashed curves).

**Figure 7:**
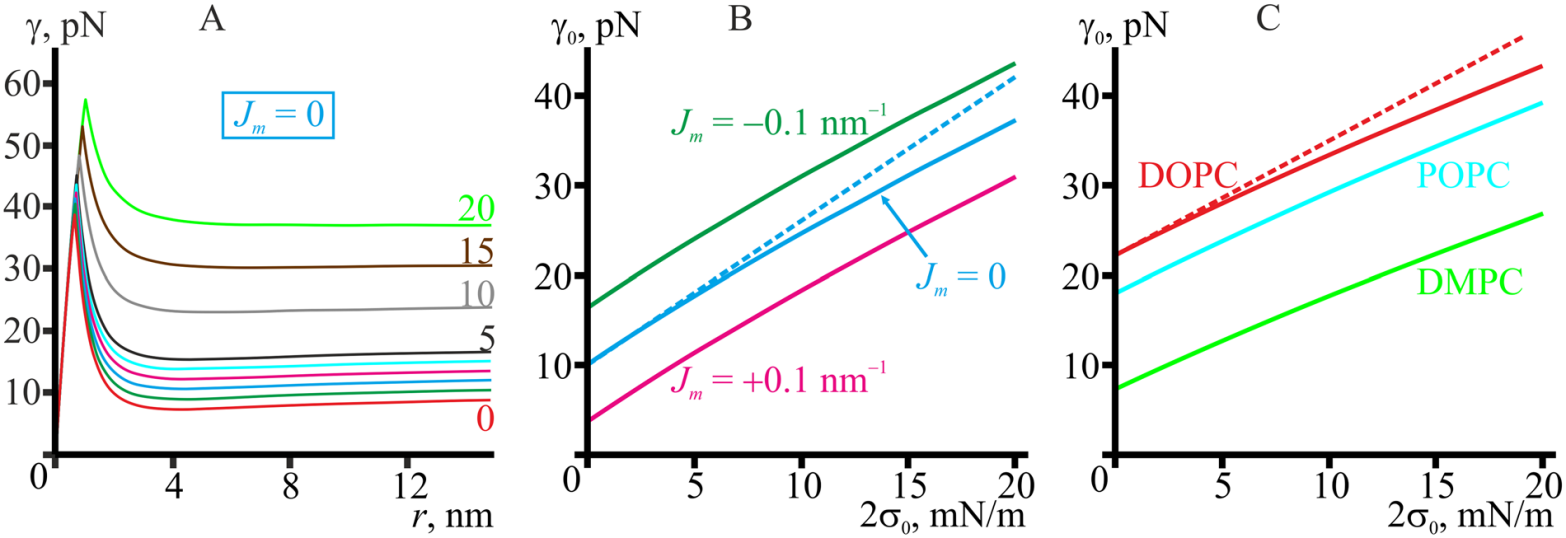
Pore line tension at different lateral tensions. (**A**) Dependence of the line tension on the pore radius at different lateral tensions applied to the membrane made of the reference model lipid (*B*_*m*_ = 8 *k*_*B*_ *T*, *K*_*A*_^*m*^ = 100 mN/m, *h*_*m*_ = 2 nm, *J*_*m*_ = 0). The membrane lateral tension, 2*σ*_0_, is specified near the curves. For green, blue, magenta and cyan curves, the lateral tensions are as follows: 2*σ*_0_ = 1, 2, 3, and 4 mN/m, respectively. (**B**) Dependence of the line tension of the pore boundary at large (infinite) radius on the membrane lateral tension, 2*σ*_0_, for model lipids: green curve for *J*_*m*_ = −0.1 nm^−1^, blue curve for *J*_*m*_ = 0, magenta curve for *J*_*m*_ = +0.1 nm^−1^. The dashed blue line is a linear approximation of the curve for the reference model lipid at small lateral tensions, obtained from Eq. (7) for Γ_0_ = 10.0 pN, *μ* = 0.807. (**C**) Dependence of the line tension *γ*_0_ of the pore boundary at large (infinite) radius on the membrane lateral tension, 2*σ*_0_, for DOPC (red curve), POPC (cyan curve), DMPC (light green curve). The dashed red line is a linear approximation of the curve for DOPC at small lateral tensions obtained from Eq. (7) for Γ_0_ = 22.2 pN, *μ* = 0.832.

Using Eq. (6), we calculated the line tension of the pore in the membrane made of the reference model lipid subjected to different lateral tensions (Fig. 7A). It turned out that the line tension indeed monotonously grew as the lateral tension increased. However, the increase of the line tension was not homogeneous: it remained almost unchanged for hydrophobic defect (*r* < 0.8 nm), increased about 1.5 times at the points of transition of the hydrophobic defect to the hydrophilic pore (peaks on curves in Fig. 7A), and further increased about 4 times for large radius, as the lateral tension increased from 0 to 2*σ*_0_ = 20 mN/m (Fig. 7A). The dependencies of the line tension *γ*_0_ for large (infinite) pore radius on the lateral tension for model lipids are presented in Fig. 7B. The curves for model lipid with different spontaneous curvatures are virtually parallel to each other, meaning that the slope of the curves should be independent on the spontaneous curvature. The dependencies of *γ*_0_ on 2*σ*_0_ for DOPC, POPC, and DMPC are presented in Fig. 7C. Here the curves for DOPC and POPC have almost the same slope, although different from the slope of the curves for model lipids, while the slope of the DMPC curve is somewhat smaller. In early studies of the pore energy landscape^13^, membrane was treated as a thick homogeneous (structureless) film with lateral tension applied; the pore boundary surface was assumed to be cylindrical. Under these assumptions, the following expression was used for the line tension: *γ*_0_ = (2 *h*)·(2*σ*_0_). For typical lateral tension of Mueller-Rudin membranes (black lipid films) of about 2 mN/m^31^ and membrane thickness 2 *h* = 4–5 nm this expression yields *γ*_0_ = 8–10 pN, which is in good agreement with the experimentally determined values for such systems^32^. However, this model predicts zero line tension at zero lateral tension, which is obviously not the case^9,11,12^. This expression for *γ*_0_ can be generalized as follows:

7 $${\gamma }_{0}={{\rm{\Gamma }}}_{0}+\mu h\cdot (2{\sigma }_{0}),$$

where Γ_0_ is the pore line tension at zero lateral tension, *μ* is a coefficient accounting for deviations of the pore boundary shape from perfectly cylindrical one (Fig. 5C). From the plots in Fig. 7B,C we obtained that for small lateral tensions *μ* = 0.807, 0.808, 0.807 for model lipid with zero, negative and positive spontaneous curvatures, respectively, and *μ* = 0.832, 0.833, 0.824 for DOPC, POPC, and DMPC, respectively. It follows that the coefficient *μ* is almost independent of the membrane thickness, spontaneous curvature, splay and lateral stretch/compression moduli, which vary in a wide range for the considered lipids. Thus, the empirical Eq. (7) with *μ*∼0.8 can serve an estimation for dependence of the pore line tension *γ*_0_ at large (infinite) radius on the applied lateral tension, although limited by only small 2*σ*_0_, as the curves *γ*_0_(2*σ*_0_) deviate from straight lines for large lateral tensions (Fig. 7B,C compare dashed and solid lines), meaning that higher-order non-linear terms on 2*σ*_0_ should be taken into account. Visually, the deviation is relatively small for 2*σ*_0_ < 7 mN/m (Fig. 7B,C).

### Membrane electrical breakdown

In this section, we analyze voltage-induced pore formation, assuming zero initial lateral tension for the sake of simplicity. In this case, the main driving force for pore formation is a large difference in the dielectric permittivity of water (*ε*_*w*_ ≈ 80) and lipid membrane (*ε*_*m*_ ≈ 2). The membrane in the electric field can be considered as a planar capacitor, tending to increase its capacitance in order to minimize electric energy. The electric field pushes water into the membrane to substitute the medium with *ε*_*m*_ ≈ 2 by the medium with *ε*_*w*_ ≈ 80, thus creating effective pressure applied to the pore edge. Classical theory of electroporation describes this phenomenon as formation of hydrophilic ion conducting defects in the membrane under applied electric field^33^. Although the formalism of the classic model is developed for thin films without internal structure, it yields reasonably good predictions in a wide range conditions used in biomedical applications^34^. This theory neglects individual structural aspects of lipid molecules, as well as the effects of the electrical double layer and water structure near the lipid headgroup dipoles. All these effects could contribute to the energy of pore formation and stability of the conducting defect^35^. However, the influence of electrical double layer is determined by the Debye length and the surface charge density on the membrane. In the present study, we limited ourselves to considering neutral lipids (phosphatidylcholines) under physiological conditions. In this case the double layer effects near the pore edge can be neglected^35^. Even if we take into account that dielectric permittivity in the region of headgroup dipoles differs from that of the bulk solution and the hydrophobic part of the membrane^36^, the hydrophobic part of the membrane having the smallest specific capacitance will still determine the total electrical capacitance of the system.

The work of lateral pressure associated with the increase of the pore radius results in decrease of the system energy proportional to the pore lumen area. The lateral pressure, as opposed to the lateral tension of the membrane, does not affect line tension of the pore boundary. Indeed, membrane deformations always increase the area of monolayer neutral surface (see Fig. 6B, dashed curves), thus requiring additional lipid to be pulled from the reservoir to the pore boundary. If lateral tension is applied to the membrane, creating additional area requires some work to be performed against the lateral tension. However, if the electric lateral pressure is applied at zero lateral tension, additional area can be drawn to the pore boundary without performing such work.

To obtain the equation for the lateral pressure caused by the electric potential *U*, we first consider the energy of substitution of a part of the membrane of a certain area *A*_0_ having the dielectric permittivity of *ε*_*m*_ with water having the dielectric permittivity of *ε*_*w*_. The membrane is considered as a planar capacitor. The change of electric energy in the process of substitution of a part of the planar capacitor of the capacitance *C*_1_ (membrane) by that with the capacitance of *C*_2_ (water) is given by:

8 $${\rm{\Delta }}{E}_{el}=\frac{{C}_{2}{U}^{2}}{2}-\frac{{C}_{1}{U}^{2}}{2}.$$

under the assumption that the substitution of a small patch of the membrane by water does not substantially alter the spatial distribution of electric potential. The equilibrium electric charges of the two capacitors are different: the initial equilibrium charge is *Q*_1_ = *C*_1_ *U*, and the equilibrium charge after the substitution is *Q*_2_ = *C*_2_ *U*. This means that we should take into account the work performed the voltage generator moving the charge:

9 $${\rm{\Delta }}{E}_{gen}=-U({Q}_{2}-{Q}_{1})=-{U}^{2}({C}_{2}-{C}_{1}).$$

The total energy change in the process of capacitor substitution is given by the expression:

10 $${\rm{\Delta }}E={\rm{\Delta }}{E}_{el}+{\rm{\Delta }}{E}_{gen}=-\frac{{U}^{2}}{2}({C}_{2}-{C}_{1}).$$

Assuming that the two capacitors differ only by the value of dielectric permittivity, we obtain that *C*_2_ = *ε*_*w*_/*ε*_*m*_·*C*_1_ = *ε*_*w*_/*ε*_*m*_·*C*_*m*_ *A*_0_, where *C*_*m*_ ≈ 0.85 μF/cm^2^ is the specific electric capacitance of the membrane^37^. Thus,

11 $${\rm{\Delta }}E=-\frac{{A}_{0}{C}_{m}{U}^{2}}{2}(\frac{{\varepsilon }_{w}}{{\varepsilon }_{m}}-1).$$

The lateral pressure caused by the electric potential *U* can thus be expressed as^33^:

12 $${p}_{l}=-\frac{\partial ({\rm{\Delta }}E)}{\partial {A}_{0}}=\frac{{C}_{m}{U}^{2}}{2}(\frac{{\varepsilon }_{w}}{{\varepsilon }_{m}}-1).$$

**Figure 8:**
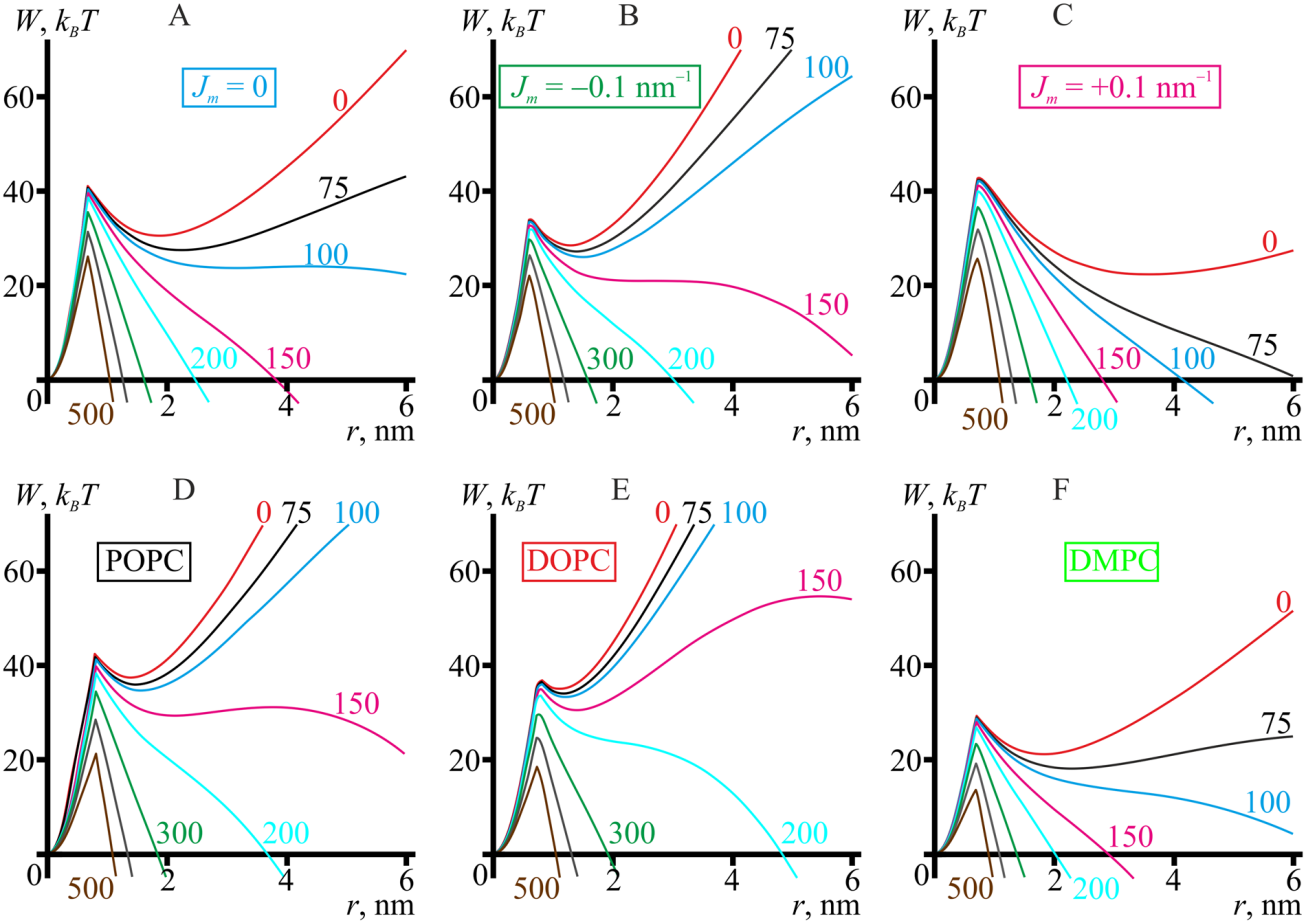
Pore formation trajectories for different transmembrane potentials. (**А**) the reference model lipid (*B*_*m*_ = 8 *k*_*B*_ *T*, *K*_*A*_^*m*^ = 100 mN/m, *h*_*m*_ = 2 nm, *J*_*m*_ = 0); (**B**) the model lipid with *J*_*m*_ = −0.1 nm^−1^; (**C**) the model lipid with *J*_*m*_ = +0.1 nm^−1^; (**D**) POPC; (**E**) DOPC; (**F**) DMPC. The values of membrane potential are (from the uppermost curves down): 0, 75, 100, 150, 200, 300, 400, and 500 mV.

Pore formation trajectories under applied electric potential can be obtained taking the trajectories calculated in the previous article^15^ for zero lateral tension and adding the work of lateral pressure, −*πr* ^2^ *p*_*l*_. The trajectories for different transmembrane potentials for model lipids with different spontaneous curvatures, DOPC, POPC, and DMPC are presented in Fig. 8.

As in the case of applied lateral tension, there are two energy barriers on the trajectories of voltage-induced pore formation at small transmembrane voltage (e.g. magenta curve in Fig. 8E). The first energy barrier at *r*∼1 nm, corresponds to the transition from a hydrophobic defect to a hydrophilic pore. After surmounting the barrier, the system finds itself in a metastable state corresponding to the local minimum of the energy at *r*∼1.5–2 nm. Formation of a supercritical (large) pore, the energy of which monotonously decreases as its radius grows, requires surmounting the second energy barrier. As can be seen in Fig. 8, the applied voltage causes abrupt decrease of the height of the second energy barrier, which vanishes at *U*∼100–200 mV. The height of the first energy barrier decreases much slower. At the transmembrane potential of *U* = 500 mV, the energy barrier to formation of supercritical pore is 26 *k*_*B*_ *T* for the reference model lipid with zero spontaneous curvature, 21 *k*_*B*_ *T* for POPC, 17 *k*_*B*_ *T* for DOPC, and 13.5 *k*_*B*_ *T* for DMPC. In the work ref.^27^ it is shown that a rate-limiting barrier of Δ*W*∼40 *k*_*B*_ *T* height can be crossed at the expense of thermal fluctuation energy within ∼1 minute under the assumption of Arrhenius dependence of the average waiting time, *τ*, of crossing an energy barrier of the height Δ*W*:

13 $$\tau =\frac{1}{\nu }{e}^{\frac{{\rm{\Delta }}W}{{k}_{B}T}},$$

where *ν* is the characteristic frequency of the attempts to cross the barrier, taken in the work ref.^27^ to be equal to the frequency of monolayer out-of-plane oscillation, about 10^13^ s^−1^. Thus, the barriers Δ*W*∼15–30 *k*_*B*_ *T* obtained for the applied voltage of 500 mV (Fig. 8) should be crossed *e* ^(40–15)^−*e* ^(40–30)^ = *e* ^25^−*e* ^10^ times faster for the same value of the frequency *v*, consistently with the available experimental data^7,13^. Similar to the case of tension-induced pores, the energy barrier for electric breakdown is lower for the lipid with negative spontaneous curvature (DOPC) than for zero spontaneous curvature lipids (POPC, model lipid with *J*_*m*_ = 0), despite relatively high line tension of the pore boundary in case of negative spontaneous curvature.

## Discussion

We have developed a model of hydrophilic pore formation induced by an external stress, such as lateral tension or transmembrane electric potential. The energy of the pore boundary was calculated with the use of liquid crystal elasticity theory adapted to lipid membranes^16^. The main criterion of adequacy of the assumptions made is agreement of the results with the experimental data.

One of the key parameters of membrane stability with respect to pore formation, line tension of the pore boundary, was found to depend both on the elastic parameters of the membrane and on the pore radius (Fig. 7). Therefore, depending on the experimental technique, different values of the line tension can be obtained for membranes of identical compositions. Supercritical pore formation requires crossing two energy barriers separated by an energy minimum (Figs 3,4 and 8). The first energy barrier at *r*∼1 nm corresponds to formation of hydrophilic pore from hydrophobic defect. The local minimum corresponds to a metastable state where principal curvatures (negative equatorial and positive meridional) of the pore boundary surface optimally compensate each other. The second energy barrier corresponds to formation of supercritical (large) pore, which energy monotonously decreases as its radius growing. The heights of the barriers depend on the elastic parameters of the membrane and on the external effects causing pore formation. We considered two such effects — application of lateral tension and of electrostatic potential to the membrane. In case of membrane electrical breakdown, the height of both barriers, and therefore the average waiting time to the occurrence of a pore, decrease monotonously under increasing transmembrane potentials (Fig. 8) until the barriers can be crossed at the expense of thermal fluctuation energy. We consider as the main driving force of pore formation the large difference in the dielectric permittivity of water and lipid membrane. Classical theory of electroporation is developed for thin films without internal structure^33^, neglecting individual structural aspects of lipid molecules, as well as the influence of electrical double layer and water structure near lipid headgroup dipoles. Thus, the line tension of the pore is assumed constant in this theory^33^. However, here we show that line tension of the pore boundary is a function of pore radius (Fig. 7). It therefor appears that a general revision of electroporation theory, not limited to dipole effects and influence of the electrical double layer, is required. We plan to undertake it in the future. In fact, re-orientation of lipid headgroup dipoles should act similar to a change of spontaneous curvature^35^, the effects of which are considered in the present (Figs 3 and 7) and in the previous article^15^. The obtained values of transmembrane voltage characteristic for pore formation (∼300 mV) are about an order of magnitude higher than the typical values of cells transmembrane potential difference (∼40–70 mV)^38^, meaning very low probability of spontaneous membrane electroporation under physiological conditions without external stress. It reflects the main natural function of cell membranes — to serve as an effective barrier between the cytosol and the extracellular milieu.

Application of lateral tension leads to a more complex behavior of the pore energy compared to the case of electrical breakdown. Under lateral tension, the height of the second barrier decreases rapidly, whereas the height of the first one grows slowly (Figs 3 and 4). The highest (rate-limiting) of the two barriers as well as the waiting time to pore occurrence are, therefore, non-monotonous functions of the applied lateral tension, each of them having a local minimum at a certain lateral tension. The optimal lateral tension values calculated for DOPC, POPC and DMPC are in agreement with the experimental data on unilamellar vesicle breakdown under slow increase of lateral tension^8^.

Such a complex dependence of the pore energy profile on the lateral tension is due to the fact that the line tension of the pore boundary is determined by elastic deformations, the energy of which grows monotonously under applied lateral tension (Fig. 7). In early attempts of pore energy landscape analysis, in which membranes were treated as thick homogeneous films, line tension value was directly estimated as *γ*∼2*σ*_0_ *h*. Under lateral tension of ∼2 mN/m, typical of Mueller-Rudin planar bilayers^13^, this estimate yields *γ* = 8–10 pN, in good agreement with the values experimentally obtained in these model systems^39,40^. However, such a simple model of the membrane fails to explain the dependence of line tension on the lipid spontaneous curvature. Besides that, the model yields *γ* = 0 at *σ*_0_ = 0, which is inconsistent with the bulk of experimental evidence^9,11,12^ as well as with results of our molecular dynamics simulations of pore closure trajectories (see the previous article^15^). In this numerical experiment, the lateral tension was zero, but the line tension of the boundary was obviously positive since it constituted the driving force causing the pore boundary perimeter to decrease^12^. To the best of our knowledge, the dependence of line tension on the lateral tension was never taken into account even in more recent studies dedicated to investigation of membrane rupture kinetics that have become classical. Earlier we predicted monotonous increase of line tension under lateral tension applied to the membrane for the boundaries of liquid-ordered membrane domains, known as rafts^30,41^. Such a dependence of line tension on surface tension is circumstantially supported by the experimentally observed growth of domain size under applied lateral tension^42,43^. Line tension increase makes domain fusion more favorable as it is associated with a decrease of the boundary length of the ensemble of domains, and hence a decrease of the total boundary energy. When monolayer deforms, its neutral surface area becomes greater than the projection area (Figs 5B and 6B), and additional work has to be performed to pull from the lipid inventory reservoir the lipid material needed to create the additional surface, thus causing line tension to increase.

We used the pore radius in the equatorial plane *r* and the half-height of the hydrophobic belt *L* as generalized coordinates defining the state of a pore. Minimizing the energy with respect to *L* under each fixed value of *r*, we obtained the optimal trajectory of pore formation from an intact bilayer through a hydrophobic defect (Fig. 2). Importantly, the obtained counterintuitive dependence of the pore energy on the applied lateral tension is not an artifact induced by the choice of coordinates or boundary conditions. In principle, a latent dependence between the coordinates or a boundary condition maintaining an unrealistic relation between certain parameters can tacitly introduce any amount of energy into the system and thus affect the calculated trajectories. Nevertheless, whatever the trajectory might be, it has to start from an intact bilayer, end at a hydrophilic pore of a sufficiently large radius, and pass through the state of the hydrophilic pore with the radius of 1 nm < *r* < 2.5 nm. In this range, the pore surface area is in any case larger than its luminal area (Fig. 6B), i.e. for forming such a pore additional amount of lipid needs to be drawn from a reservoir and hence work has to be performed against the lateral tension. On the trajectory we have analyzed, this work is minimal since we have optimized the shape of the pore boundary explicitly taking into account the applied lateral tension (Fig. 5C). Therefore, the non-monotonous dependence of the pore energy on the applied lateral tension is to be even more pronounced for other trajectories.

Besides, the observed dependence of energy barriers on the lateral tension is not a consequence of a particular model of hydrophobic defect. Indeed, at the stage of hydrophobic defect the pore lumen radius is relatively small, and thus the term −*πr* ^2^ *σ*_0_ corresponding to the work of the lateral tension is negligible. At small radii, the surface tension of the interface between water and lipid tails, *σ*_*h*_, is low, as follows from Marcelja theory^19^. In accordance with Table 1, change of the hydrophobic belt height is the softest mode of deformation. This means that at the stage of hydrophobic defect the area of the neutral surface of a monolayer can be maintained practically constant by varying the height of the hydrophobic belt; the adjustment costs small amount of energy due to low *σ*_*h*_. For this reason, the energy of the hydrophobic defect is almost independent on the lateral tension up to the tension values as high as 2*σ*_0_∼20 mN/m (Figs 3 and 4).

**Figure 9:**
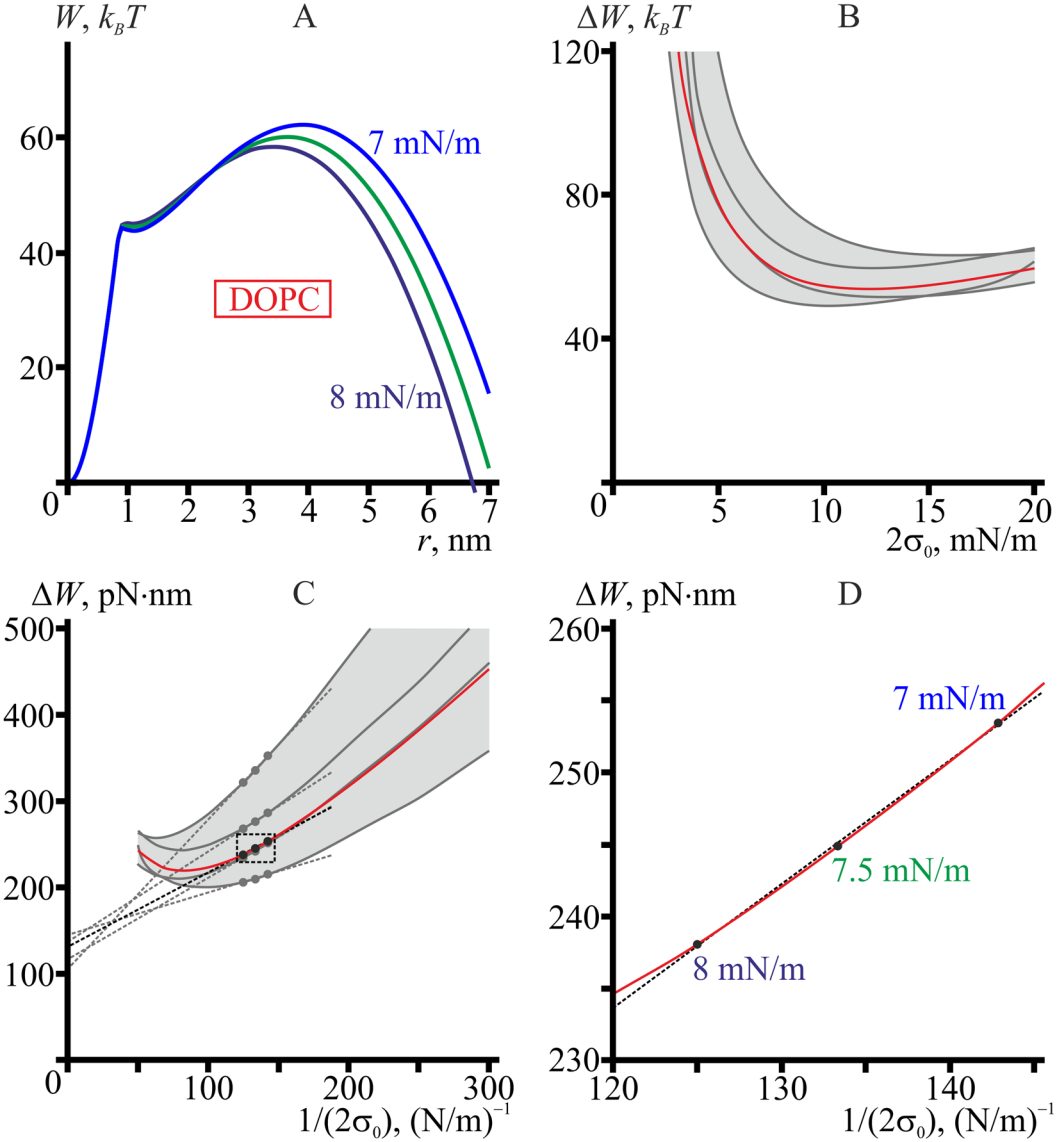
(**A**) Dependencies of energy of the pore formed in DOPC membrane on the pore radius for lateral tension of 2*σ*_0_ = 7 mN/m (blue curve); 7.5 mN/m (green curve); 8 mN/m (dark blue curve). (**B**) Dependence of the calculated maximal energy barrier, Δ*W*, for DOPC on membrane lateral tension, 2*σ*_0_ (the same curves as in Fig. 4B plotted to larger scale). (**C**,**D**) Dependence of the calculated maximal energy barrier, Δ*W*, for DOPC on the inversed membrane lateral tension, 1/(2*σ*_0_). Black and grey circles correspond to membrane lateral tensions 7, 7.5, and 8 mN/m, applied to GUVs in the experiment of the article ref.^47^ to determine the activation energy of pore formation. Dashed lines are linear fits of the calculated maximal energy barriers at these three tensions. Panel D is a magnification of the region of the panel C outlined by dashed black rectangle. In panels B–D, the red curves correspond to the average values of experimentally determined elastic parameters for DOPC (*B* = 10.3 *k*_*B*_ *T*; *K*_*A*_ = 133 mN/m; *h* = 1.45 nm; *J*_*DOPC*_ = −0.091 нм^−1^); the grey curves correspond to different sets of the elastic parameters combined from maximal and minimal parameter values within experimentally determined confidence interval, as indicated in the section “System parameters”. The grey curves bound the possible variance of the dependencies Δ*W*(2*σ*_0_) and Δ*W*((2*σ*_0_)^−1^), shown as light grey zones in panels B,C.

Recently, it was found that rupture of giant unilamellar vesicles under applied lateral tension can be considered as an exponential decay process with a rate constant *k*_*p*_ dependent on the lateral tension^28,44–46^. For all the tested lipids (including DOPC), the rate constant monotonously increases with the lateral tension. This is equivalent to monotonous decrease of the average waiting time of GUV rupture at increasing lateral tension. Under assumption of Arrhenius dependence of the average waiting time to cross an energy barrier on its height (Eq. (13)), it means that the effective (i.e. maximal) energy barrier to pore formation also decreases. It is what we have obtained for DOPC, the lipid used in our calculations and a number of experiments: the maximal energy barrier monotonously decreases up to the tensions of 2*σ*_0_∼12 mN/m (Fig. 4B, red curve). The dependence of the maximal barrier, Δ*W*, on the lateral tension is presented in Fig. 9B. For larger lateral tensions (12 mN/m < 2*σ*_0_ < 20 mN/m), the barrier grows. Practically, such large tensions cannot be statically applied to GUV membranes. In the papers of E. Evans and co-workers^8,28^, constant tension ramp was applied, causing GUV rupture at the tensions of up to some 35 mN/m for the most resilient lipid (diC22:1) at the highest loading rates of 124 mN/m/s^8^. The determined *k*_*p*_ values monotonously increased with the increasing *σ*_0_ in the entire range of tensions^28^, and the average rupture tensions increased with the increasing loading rate^8,28^. However, at high loading rates one could expect that the tension would be non-uniformly distributed over the GUV membrane, i.e. tension spatial gradients can occur. The pore can appear at any location on a GUV, so one cannot unambiguously conclude that the particular pore was formed exactly at the measured tension of GUV rupture. Besides, the tension gradient itself might alter the process of pore formation compared to the case of statically applied lateral tension, since the gradient may cause local viscous lateral flows of lipid. For low loading rates, the DOPC GUV rupture occurred most frequently at the tension of about 8 mN/m (ref.^8^) This value correlates well with the substantial drop of the maximal energy barrier calculated in the present work (Figs 4B and 9B): further increase of the lateral tension up to 2*σ*_0_∼12 mN/m causes only a slight decrease of the barrier due to proximity of the local minimum.

Based on the temperature dependence of the rate constant, *k*_*p*_, the activation energy *U*_*a*_ of tension-induced DOPC GUV rupture^47^ appears to be about 20 *k*_*B*_ *T*. In the experimentally tested range of lateral tensions of 7–8 mN/m, the activation energy is found to be a linear function of the inversed lateral tension, i.e. in our notation:

14 $${\rm{\Delta }}W={U}_{0}+\frac{\pi {\gamma }_{0}^{2}}{(2{\sigma }_{0})}=19{\rm{pN}}\cdot {\rm{nm}}+\frac{\pi {(11.6{\rm{pN}})}^{2}}{(2{\sigma }_{0})}.$$

The line tension *γ*_0_ = 11.6 pN is assumed independent of *σ*_0_. *U*_0_ is a fitting constant found to be about 19 pN·nm∼5 *k*_*B*_ *T*, the physical meaning of which is the activation energy at infinite lateral tension, as seen from Eq. (14). To compare the data obtained in the work ref.^47^ with the results of our calculations, we re-plotted the maximal energy barrier Δ*W*(2*σ*_0_) for DOPC (Fig. 9B) as a function of the inversed lateral tension, i.e. Δ*W* vs. 1/(2*σ*_0_) (Fig. 9C), and changed the units of energy from *k*_*B*_ *T* to pN·nm, to match those used in the article ref.^47^. The black circles in Fig. 9C,D correspond to membrane lateral tensions of 7, 7.5, and 8 mN/m applied to GUVs in the experiments reported in the article ref.^47^ to determine the activation energy of pore formation. The calculated curve of the maximal energy barrier in the range of lateral tensions 7–8 mN/m is almost indistinguishable from the linear regression

15 $${\rm{\Delta }}W=130.2{\rm{pN}}\cdot {\rm{nm}}+\frac{\pi {(16.6{\rm{pN}})}^{2}}{(2{\sigma }_{0})},$$

calculated for three lateral tensions of 7, 7.5, and 8 mN/m (Fig. 9D, compare solid red and dashed black lines). This conclusion agrees qualitatively with the results obtained in the work ref.^47^. However, the calculated dependence Δ*W* vs. 1/(2*σ*_0_) in the entire range of lateral tensions deviates substantially from the straight line defined by Eq. (15) (dashed black line in Fig. 9C), implying that the model proposed for dependence of the activation energy on lateral tension in the works refs^46,47^ is oversimplified, although adequate for the experimentally addressed range of lateral tensions. Our results agree qualitatively with the data of the article ref.^47^ in the range of lateral tensions 7–8 mN/m, but there is substantial quantitative discrepancy in the values of activation energy (compare Eq. (14) and Eq. (15) at equal lateral tensions). The main reason for such discrepancy is the difference of line tension values calculated in the framework of our model and determined experimentally in the work ref.^47^. Namely, these values differ by a factor of about 1.5 (16.6 pN vs. 11.6 pN), leading to more than 2 times different activation energy due to its quadratic dependence on line tension. However, both values (16.6 pN and 11.6 pN) are well within the range of 3.9–27.7 pN experimentally determined for DOPC by different techniques, as reviewed in ref.^11^, so the discrepancy does not constitute a direct contradiction. The measured values of line tensions depend on the particular method of model membrane formation, on the type of the external stress applied to form the pore, and even on the lipid manufacturer. Specifically, the line tension is predicted to drop substantially if the membrane contains even a small amount of hydrophobic or weakly polar organic impurity^5,6^. Such impurities are hypothesized to accumulate in the rim around the pore edge between two monolayers of membrane, allowing partial relaxation of membrane curvature. This conclusion correlates well with the small values of pore line tension (∼6 pN) measured on black lipid membranes containing decane as organic solvent for lipids^7,13^.

In order to calculate the energy barriers of pore formation, we utilized experimentally determined values of the elastic parameters. These values are known with finite uncertainties, which are presented for DOPC membranes in the section “System parameters”. We combined different limiting values of the elastic parameters — the upper and lower limits of the experimental confidence interval — to distinct sets and calculated the dependencies of rate-limiting energy barrier, Δ*W*, on the applied lateral tension (Fig. 9B, grey curves) and inverse lateral tension (Fig. 9C, grey curves) for DOPC membrane. The curves generated for different sets of the elastic parameters form a spectrum of possible dependencies Δ*W*(2*σ*_0_) and Δ*W*((2*σ*_0_)^−1^) (Fig. 9B,C, shown in light grey). The linear regressions of the dependencies Δ*W*((2*σ*_0_)^−1^), calculated for the different sets of the elastic parameters yields (Fig. 9C, grey dashed lines):

16 $${\rm{\Delta }}W=(105.3-144.3){\rm{pN}}\cdot {\rm{nm}}+\frac{\pi {((23.4-12.5){\rm{pN}})}^{2}}{(2{\sigma }_{0})}.$$

The experimentally determined activation energies are compared with the calculated values in Table 2.

**Table 2:** Experimentally determined activation energies of pore formation in DOPC membranes at different lateral tensions (ref.^47^) compared with the height of the energy barrier, calculated in the framework of continuum theory.

	Activation energy, ∆*W*, pN·nm/*k*_*B*_ *T*			Linear regression parameters	
	7 mN/m	7.5 mN/m	8 mN/m	*U*_0_, pN·nm/*k*_*B*_ *T*	*γ*_0_, pN
Data of ref.^47^	81.5 ± 1.9	77.1 ± 1.4	73.7 ± 1.9	19 ± 3	11.6 ± 0.2
	19.8 ± 0.5	18.8 ± 0.4	17.9 ± 0.5	4.7 ± 0.6	
Calculated	253.4	244.9	238.1	130.2	16.6
average	62.3	60.1	58.5	32	
Calculated	215–352	210–335	206–321	105.3–144.3	12.5–23.4
scattered	50.6–86.5	51.5–82.4	52.7–78.9	25.9–35.4	

Calculated average — values obtained for *B* = 10.3 *k*_*B*_ *T*; *K*_*A*_ = 133 mN/m; *h* = 1.45 nm; *J*_*DOPC*_ = −0.091 нм^−1^. Calculated scattered — values obtained for different sets of elastic parameters, combined from those taken within the experimental confidence intervals, as indicated for DOPC in the section “System parameters”.

Apart from possible different values of line tension, relatively low activation energies determined in the work ref.^47^ can result from the specifics of the experimental system. Generally, lateral tension is applied to GUV membrane by partial aspiration through a micropipette. In order to make GUVs visible in a phase contrast microscope, they are formed in sucrose solution and then transferred to glucose solution of similar osmolarity, having a different refractive index^8,11,28,39,44–47^. The practically used sucrose and glucose concentrations vary from 100 mM^44–47^ to 200–260 mM^8,11,28^ (in some cases it is as high as 550 mM^39^). In the work ref.^47^, GUVs were initially held to the micropipette by a small hydrostatic pressure generating the lateral tension of ∼0.5 mN/m, which is a widely used procedure allowing to release the so-called hidden area of the membrane^48^. After 2 minutes, the tension was rapidly increased to the desired value (7–8 mN/m) and maintained constant until GUV rupture. It is possible that at the stage of initial attachment, before application of the large lateral tension, a transient pore (either hydrophilic or hydrophobic) of some radius stochastically formed. Due to dependence of the surface tension of a hydrophobic cylinder filled with water on the cylinder radius revealed by Marcelja theory^19^, the pore energy depends on pore radius quadratically at small radii (Figs 3 and 4). Because of that, for example, for DOPC formation of the hydrophobic defect of 0.7 nm diameter requires less than 10 *k*_*B*_ *T* of energy (Fig. 4A). Such defects should stochastically form in the membrane with high frequency. In the presence of a pressure drop across the membrane, viscous water/sucrose solution would flow through the defect outward of the GUV. The viscous flow can deform the planar membrane around the pore to a cone-like shape and stabilize the pore at some radius; indeed, stabilization of macroscopic pores in GUVs by viscous flow was used for determining the pore line tension in the work ref.^39^. At least, the flow can decrease the probability of pore closure, thus increasing its characteristic lifetime. This should shift the reference state with respect to which the activation energy is measured, resulting in systematic underestimation of the energy barrier of supercritical pore formation.

Another possible explanation of the quantitative discrepancy between the activation energies determined experimentally in the ref.^47^ and those calculated in the present work is the presence of the metastable state of the pore, characterized by the local minimum of the pore energy at *r*∼1.1 nm (Fig. 9A). Although the energy barrier of transition to the metastable state is quite high (about 40 *k*_*B*_ *T*, dashed curve Δ*W*_1_(2*σ*_0_) in Fig. 4B), the barrier depends on the lateral tension very weakly, meaning that the probability of metastable pore formation is also almost independent on the lateral tension. Once formed, the metastable pore may be dynamically stabilized against rapid re-closure by viscous flow through it. The defects with relatively long lifetime were observed in a number of studies of pore formation processes^10,13,39^, directly as long-term fluctuations of membrane electric conductance^10,13^ or indirectly as decrease of critical rupture tension of preliminary porated and resealed vesicles as compared to intact ones^39^. We thus hypothesize that in the works refs^44,47^ the authors might determine the activation energy of supercritical pore formation not with respect to the ground state occupied by numerous hydrophobic defects of small radii (*r*∼0), but rather with respect to the metastable state occupied by a few metastable hydrophilic pores (*r*∼1.1 nm) in each vesicle. (Note that the luminal diameter of a metastable pore in DOPC membrane is about 2·(1.1–0.7) = 0.8 nm only, where 1.1 nm is the radius of neutral surface of the membrane monolayer, and 0.7 nm is an approximate thickness of DOPC polar head.) In other words, possibly the parameter determined in the works refs^44,47^ is not the maximal barrier Δ*W*_2_ (transition from intact bilayer to supercritical pore), but the barrier Δ*w*_2_ for transition from the metastable state to the supercritical pore (Fig. 3A). The activation energies of the latter transition, calculated for DOPC in the framework of the continuum theory, are 18.4, 15.6, and 13.4 *k*_*B*_ *T* for 2*σ*_0_ = 7, 7.5, and 8 mN/m, respectively (dotted curve Δ*w*_2_(2*σ*_0_) in Fig. 4B), which is in much better agreement with the experimental results of the work ref.^47^.

## Conclusions

We have developed a theoretical model of the pore formation in membrane subjected to external stress: applied lateral tension or transmembrane voltage. The proposed model explicitly takes into account the optimal shape of the pore surface obtained by energy minimization for each state of pore formation and growth, and thus provides a more physically grounded description of the pore formation phase trajectory than can be obtained with any postulated shape of the pore boundary. The model predicts qualitatively different behavior of directly measurable parameters, such as waiting time till pore formation or membrane lifetime, for the pores induced by mechanical lateral tension and transmembrane electric potential difference. It also explains significant spread of the values of such pore parameters as line tension of the boundary reported by different groups in the apparently identical conditions. The hitherto disregarded dependence of the boundary line tension on the membrane surface tension and on pore radius make the measured value a function of the measurement method and of a number of additional parameters traditionally not controlled in the experiments. The level of resolution of the predictions of the model at the currently attained level of uncertainty of measurement of the required input parameters is sufficient to capture the differences between biologically significant lipid species and experimental conditions. One of the key parameters characterizing stability of the membrane with respect to pore formation, line tension of the pore boundary, was found to increase with application of the lateral tension, meaning that classic Derjaguin equation of energy of the pore in a thin structureless film, Eq. (1), should be generalized as:

17 $$E(r)=2\pi r\gamma (r,{\sigma }_{0})-\pi {r}^{2}{\sigma }_{0}.$$

Because of such dependence of the line tension on the lateral tension, the average waiting time of pore formation under statically applied lateral tension is predicted to be a non-monotonous function of the lateral tension value. On the contrary, the line tension was found to be independent on the applied transmembrane voltage, thus leading to monotonous decrease of the expected waiting time of membrane electric breakdown on the applied voltage. In future, for general theoretical description of the process of transverse pore formation, we plan to extend the developed model to explicitly address asymmetric systems with pressure gradient across the membrane.
